# High-tannin food enhances spatial memory and scatter-hoarding in rodents via the microbiota-gut-brain axis

**DOI:** 10.1186/s40168-024-01849-2

**Published:** 2024-07-29

**Authors:** Xiangyu Zhao, Jiawei Guo, Yiming Wang, Xianfeng Yi

**Affiliations:** 1 School of Life Sciences, Qufu Normal University, Qufu, 273165 China; 2 Present address: Huxi Middle School, Dongchangfu District, Liaocheng, 252000 China

**Keywords:** Tannin, Hoarding rodents, Spatial memory, Microbiota-gut-brain axis

## Abstract

**Background:**

The mutually beneficial coevolutionary relationships between rodents and plant seeds have been a theme of research in plant-animal relationships. Seed tannins are important secondary metabolites of plants that regulate the food-hoarding behavior of rodents; however, the underlying molecular mechanisms are not yet clear. In this study, we investigated whether and how seed tannins improve spatial memory and regulate the hoarding behavior of *Tamias sibiricus* by altering their gut microbiota.

**Results:**

We showed that acorn tannins not only improved spatial memory but also enhanced scatter-hoarding in *T. sibiricus*. Changes in the composition and function of the gut microbiota in response to tannins from acorns are closely related to these improvements. Metabonomic analyses revealed the role of gut isovaleric acid and isobutyric acid as well as serum L-tryptophan in mediating the spatial memory of *T. sibiricus* via the gut microbiota. The hippocampal proteome provides further evidence that the microbiota-gut-brain axis regulates spatial memory and scatter-hoarding in animals. Our study is likely the first to report that plant secondary metabolites improve hippocampal function and spatial memory and ultimately modulate food-hoarding behavior via the microbiota-gut-brain axis.

**Conclusion:**

Our findings may have resolved the long-standing puzzle about the hidden role of plant secondary metabolites in manipulating food-hoarding behavior in rodents via the microbiota-gut-brain axis. Our study is important for better understanding the mutualistic coevolution between plants and animals.

Video Abstract

**Supplementary Information:**

The online version contains supplementary material available at 10.1186/s40168-024-01849-2.

## Background

Seed dispersal by food-hoarding animals plays a key role in plant regeneration and the population dynamics of various plants bearing large seeds [1–3]. Scatter-hoarding animals usually bury seeds in small shallow pits scattered within their home range [4, 5]. Although the specific mechanism of seed dispersal by scatter-hoarding animals is still unclear, scatter-hoarding behavior is crucial for seed dispersal and seedling recruitment of large-seeded species in forest ecosystems [6]. Previous studies have shown that food-hoarding animals rely mainly on spatial memory to cache and recover their cached seeds [7, 8]; therefore, spatial memory appears to be a decisive factor in determining how the seeds are cached and then retrieved. In this scenario, any biotic or abiotic factors that can affect spatial memory will determine the fitness of seed dispersal, as well as the mutualistic relationship between seeds and food-hoarding animals.

Tannins, one of the most common secondary metabolites that exist widely in various plant organs, such as fruits and seeds [9], have negative and even toxic effects on the growth and metabolism of seedeaters due to their ability to bind proteins or interact with carbohydrates, lipids, and metal ions [10, 11]. Despite its toxicity, food-hoarding animals prefer to cache high-tannin food over those containing a low level of tannins, which has been termed the “high tannin hypothesis” [10, 12–14]. The fact that the hoarding process and storage duration cannot reduce tannin toxicity in cached seeds implies that food-hoarding animals may benefit more from the high level of seed tannins than ever thought. An increasing body of literature has shown that tannins have the potential to prevent neural damage in the hippocampus and hippocampal-dependent cognition [9, 15, 16]. Although the underlying mechanism remains unclear, alteration of the gut microbiota by tannins is expected to play a hidden role through the gut-brain axis.

The growing power and finesse of metagenomics studies have quickly expanded our knowledge of the importance of the gut microbiota in mutualistic relationships with its host [17–22]. The gut microbiota plays a crucial role not only in the development and functional maturation of the gut immune system but also in the maintenance of animal health through the microbiota-gut-brain axis [23]. The gut microbiota can produce various essential neurotransmitters and neuromodulators, such as gamma amino acid, 5-hydroxytryptamine, dopamine, and various short-chain fatty acids, to connect the gut-brain axis and ultimately regulate brain function through different transport pathways [24–27]. Evidence showed that changes in the gut microbiome altered the performance of rodents in a series of visual-spatial learning and memory tasks, restored impairments in spatial memory behavior [28, 29], or improved episodic or long-term learning and spatial memory [30–32]. Tannins, including condensed tannins and hydrolyzed tannins, have been reported to regulate the gut microbiota composition and bacterial metabolites [33–35], which in turn affect the physiological, behavioral, and cognitive functions of animals [36, 37]. Previous studies have either explored the effects of tannin addition on cognitive ability or revealed the relationship between the gut microbiota and spatial memory in mammals [38–41]. However, few studies have bridged the gap between the effects of tannins on the gut microbiota and the improvement of spatial memory, to uncover the secondary metabolite-microbiota-gut-brain axis in the context of mutualistic interactions between plant seeds and animals.

Food-hoarding animals play a crucial role in various forest ecosystems [42, 43]. Each individual scatter-hoarding animal is estimated to store several thousand seeds in their scattered caches within a single season [44]. Acorns of *Quercus*, often containing a high level of tannins, constitute the main food items cached by various scatter-hoarding animals [45, 46]. The mutualistic relationship between acorns and scatter-hoarding rodents has been considered one of the crucial factors for maintaining the biodiversity of oak forests [47–49]. However, it remains unclear what drives the mutualistic relationship between food-hoarding animals and toxic acorns with high tannins, although the “high tannin hypothesis” points out the preference of animals for high-tannin seeds. Here, the composition and function of the gut microbiota and the spatial cognition and hoarding behavior of Siberian chipmunk, *Tamias sibiricus*, an important scatter-hoarding animal widely distributed in oak forests, were investigated to study whether and how the seed tannins of *Quercus variabilis* regulate spatial memory and hoarding behavior by using metagenomics, targeted and non-target metabolomics, proteomics, fecal microbiota transplantation (FMT), and behavioral procedures. Our goal was to explore the underlying mechanisms by which toxic tannins in acorns modify the mutualistic interactions between plants and hoarding animals from the perspective of microbiota-gut-brain axis, which is highly important for revealing coevolution among plants, gut microbiota, and their host animals. We specifically addressed the following hypotheses: (1) seed tannins not only improve the spatial memory of *T. sibiricus* but also may regulate its hoarding behavior, (2) improvement of spatial memory is closely related to alteration of the gut microbiota by tannin addition, and (3) metabolites such as neurotransmitters and short-chain fatty acids serve as hidden players in regulating the hippocampus through the microbiota-gut-brain axis. We expect that our research will pave a new way to study mutualistic relationships between animals and plants regulated by the interaction between secondary metabolites and the gut microbiota.

## Materials and methods

### Animals and handling

The Siberian chipmunks purchased from Jiaxiang Tengxiang Breeding Co., Ltd. (Jining, China) were housed separately in standard plastic cages measuring 53 cm × 39 cm at room temperature and under a natural light cycle and were allowed free access to tap water and food in this study. After 1 week of acclimatization, the chipmunks were randomly divided into four groups: the TST group (acorn addition group, *n* = 7), TSC group (control group, *n* = 7), T-tan group (tannic acid addition group, *n* = 8), and T-con group (control group, *n* = 8). The TSC and T-con groups were fed standard rat chow (Jinan Pengyue Experimental Animal Breeding Co., Ltd., Jinan, China) and purified water ad libitum. Each *T. sibiricus* individual in the TST group received standard rat chow together with five acorns of *Q. variabilis* every day for 20 days. The acorns of *Q. variabilis*, containing approximately 10% tannins, were collected from a local area in a state-owned forest in Shimenshan, Qufu, China. Individuals in the T-tan group were provided standard rat chow and 2% tannin solution prepared using tannic acid (Macklin, Shanghai, China) for a period of 10 days. After feeding, fresh feces were collected, snap frozen in liquid nitrogen, and stored at − 80 °C for subsequent 16S rDNA gut microbiota analysis. In total, six fecal samples were collected for each of the TSC and TST groups, and eight samples were collected for each of the T-con and T-tan groups. To collect feces, we confined each chipmunk into a sterilized live trap measuring 20 cm × 11 cm × 9 cm that was placed on a piece of A4 paper and allowed it to defecate. At least 10–15 pellets of fresh feces were collected from each chipmunk within 5 min.

### Hoarding behavior of T. sibiricus in the artificial enclosures

To test whether high-tannin food consumption modifies the hoarding behavior of *T. sibiricus*, we carried out food-hoarding experiments in five duplicated outdoor arenas (10 × 10 m and 2.5 m high). We paved the ground of each enclosure with bricks to create an 8 × 8 grid of 64 evenly spaced, small, shallow pits (24 × 12 cm and 6 cm deep) each separated by 1 m. The pits were filled with fine sand to allow the animals to cache, resulting in an arena that was nearly all paved, except for the small pits [50]. A seed station (0.5 m^2^) was established for seed placement in the center of each enclosure, and a man-made nest and a water plate were placed in one corner of each enclosure for *T. sibiricus*. All enclosures were well protected with a steel net roof to prevent access from potential predators [50]. The chipmunks were acclimated indoors for 1 week prior to the scatter-hoarding experiments in the enclosures. The chipmunks were individually introduced into each enclosure at 06:00 in the morning and allowed to freely carry, eat, and hoard the 20 seeds of *Castanea mollissima* placed at the seed station of the enclosure. Here, seeds of *C. mollissima* rather than acorns of *Q. variabilis* were provided to avoid any interference from feeding experience or chipmunk preferences. At 18:00, the chipmunks were removed from the enclosures, and the seed fates were checked. The seeds handled by the chipmunks were grouped into intact in situ (IIS), eaten in situ (EAR), intact after removal (IAR), scatter-hoarded in shallow pits (SH), and larder-hoarded in nests (LH).

### Eight-arm maze test

We used an eight-arm maze to test whether the consumption of tannins improved the spatial memory of *T. sibiricus* following the hoarding experiments. The 8-arm maze, 75 cm in diameter, consists of eight organic arms 28 cm in length, 6.5 cm in width, and 60 cm in height, which prevents escape of the focal animals. A camera wired to be scored in real time by ANY-maze 7.0 animal behavior video analysis system (Stoelting Co., USA) was placed 1.5 m above the center of the 8-arm maze to measure working memory error (WME), i.e., reentry into the baited arms, and reference memory error (RME), i.e., first entry into the unbaited arms. The eight-arm maze experiment was divided into three consecutive phases. In the acclimatization phase (2d), chipmunks placed in the eight-arm maze were allowed to move freely and consume the food pellets for 10 min every day. In the training phase (7d), after the adaptation phase, training was carried out once per day for 7 days. The food pellets were placed in four randomly selected arms, and the daily sequence was kept unchanged throughout the training stage. The chipmunks were first confined to the center of the maze for 30 s and then allowed to enter any arm to eat the food pellets completely. The training process was terminated if the chipmunks were unable to consume the pellets within 10 min. Between each of the two training sessions, we cleaned the maze completely using 75% alcohol to avoid odor interference. In the test phase (1 d), on the 10th day, the food pellets were removed from the arms. The chipmunks were allowed to move freely in the maze for 5 min, during which the WME and RME parameters were recorded to test the spatial memory of *T. sibiricus*.

### Fecal microbiota transplantation (FMT)

To test whether the gut microbiota participates in improving spatial memory after tannin consumption, we performed fecal microbiota transplantation in 8-week-old C57BL/6 J mice (Jinan Pengyue Experimental Animal Breeding Co., China). Mice were housed individually in polystyrene plastic cages (37 cm × 27 cm × 17 cm) where they had free access to water and standard rat chow. One week after acclimatization, the mice were randomly divided into four recipient groups: TST-FMT (*n* = 18), TSC-FMT (*n* = 18), T-tan-FMT (*n* = 13), and T-con-FMT (*n* = 12) to receive the fecal microbiota from the four donor chipmunk groups, respectively. Prior to feces collection, the chipmunks had stopped consuming acorns/tannic acid for 5 days. To collect feces pellets, each chipmunk was individually confined in a sterilized cage (L × W × H = 20 cm × 11 cm × 9 cm). At the same time, fresh feces were collected from each chipmunk within 10 min with forceps. The fresh feces (200 mg) from each donor group were mixed and dissolved in 2 mL of sterile 0.9% saline pellets. After slight shaking, the inoculum was centrifuged at 37 °C, 500 r/min for 10 min under aerobic conditions. The supernatant was then immediately collected for gavage. Each recipient mouse was administrated by gavage with 300 μl bacterial suspension from donors every day for 10 consecutive days. To ensure the freshness of the bacterial suspension, fresh feces were collected daily for gavage. During the gavage period, the mice were provided free access to water and standard rat chow ad libitum. Four weeks after FMT, the Barnes maze test was performed to test the difference in spatial memory between the recipients.

### Barnes maze test

The Barnes maze is a rotatable circular platform constructed of organic wood panels 91 cm in diameter with 20 equally spaced circular holes (5 cm × 5 cm) around the perimeter of the platform. Only one hole is connected to a dark black box (i.e., the target box), while the other holes are hollowed out. A camera from the Any-maze 7.0 Animal Behavior Video Analysis System (Stoelting Co., USA) was placed 1 m above the center of the maze to monitor the mice of the TST-FMT, TSC-FMT, T-tan-FM, and T-con-FMT groups, and the data were collected simultaneously. Before the start of each training session, the mice were placed in a rectangular box at the center of the maze to restrict movement for 5 s. Then, the box was removed, and the mice were allowed to move freely on the platform to locate the target box. The training session lasted 4 min and was terminated when the mice entered the target box with their whole body for 30 s. However, if the mice failed to locate the target box within 4 min, they were manually placed in the target box for 30 s. The maze was randomly rotated to change positions between each session to prevent the mice from relying on scent to determine the location of the target box, while the orientation of the target box was always fixed. At the same time, the maze was cleaned with 75% alcohol to avoid odor disturbance between each training session. Each mouse was trained once per day for six consecutive days. On day 7, spatial memory ability was tested with the same procedure as in the training phase. The time taken and distance traveled to locate and enter the target box were measured for each mouse. After that, the mice in the TST-FMT and TSC-FMT groups were immediately sacrificed and dissected on ice to quickly collect the cecal contents for subsequent gut microbiota assays.

### Sample collection of *T. sibiricus*

After the hoarding behavior in the enclosures and the fecal microbiota transplantation (FMT) experiment, four individuals of *T. sibiricus* from each group (i.e., TSC and TST) were randomly selected and anesthetized. The blood sample was extracted by cardiac puncture and then centrifuged to collect serum in sterile EP tubes, snap frozen in liquid nitrogen, and stored at − 80 °C for subsequent serum metabolite analysis. Then, the animals were immediately euthanized and dissected on ice to quickly collect the bilateral hippocampi and cecal contents. All samples were immediately frozen in liquid nitrogen and stored at − 80 °C for subsequent hippocampal proteomic assays, gut microbiota tests, and short-chain fatty acid assays. To avoid killing too many animals and maximize animal welfare, individuals in the T-con and T-tan groups were not sampled; these groups were expected to show patterns similar to those of the TSC and TST groups.

### Analysis of the gut microbiota

#### DNA extraction

The fecal samples were transported on dry ice to OE Biotech Shanghai for testing following the manufacturer’s instructions. A MagPure Soil DNA LQ Kit (Magen, Guangdong, China) was used to isolate bacterial DNA from feces. We determined the DNA concentration using a NanoDrop 2000 spectrophotometer (Thermo Fisher Scientific, Waltham, MA, USA) and examined the DNA integrity via agarose gel electrophoresis. A universal primer pair (343F: 5′-TACGGRAGGCAGAG-3′; 798 r: 5′-AGGGTATCTAATCCT-3′) was used. The reverse primer contains the sample barcode, and two primers are connected to the Illumina sequencing adapter.

#### Library construction and sequencing

The library was constructed and sequenced using gel electrophoresis to observe the quality of the amplicons. The PCR products were purified using Agcourt AMPure XP beads (Beckman Coulter Co., USA) and then quantified using a Qubit dsDNA detection kit. Then, the concentration was adjusted for sequencing performed on an Illumina NovaSeq 6000 with a read cycle of 250 bases per two paired ends (Illumina Inc., San Diego, CA; OE Biotech; Shanghai, China). The raw image data files obtained were transformed into raw sequences (raw data) by base call analysis.

#### Processing of sequencing data

The raw reads were processed to filter sequences for length (800–2500 bp) and quality through SMRT Portal according to the provided protocol [51]. We further filtered the sequences by removing barcodes, chimeras, primer sequences, and those containing 10 consecutive identical bases. Then, we used the unique sequences among these remaining reads to define the operational taxonomic units (OTUs) through USEARCH (version 10) with a threshold of 98.65% similarity. We used Silva Release 138.1 (http://www.arb-silva.de), employing the UCLUST algorithm (v1.2.22q) at a confidence threshold of 0.8, to determine the taxonomic identities of the phylotypes [51]. We analyzed the raw sequencing data to generate rank-abundance curves and specaccum curves and performed core microbiome analysis and distances between groups using the QIIME software package (version 1.9.0). The annotations were obtained using Trimmomatic (version 0.35), Flash (version 1.2.11), split_libraries in QIIME (version 1.8.0), UCHIME (version 2.4.2), VSEARCH software, and the RDP classifier. We used the naive Bayesian classification algorithm to obtain the annotation information for OTUs.

#### Alpha- and *beta*-diversity analyses

Alpha-diversity indices (Chao1 and Shannon) were assessed based on Mothur v.1.35.1. and compared between the animal groups using the Wilcoxon signed-rank test. Analysis of similarities (ANOSIM) was used to detect the microbial community changes (beta diversity) between the animal groups using PCoA based on the unweighted UniFrac distance matrix and Bray–Curtis distance matrix with a 999 permutation *t*-test in R (Vegan 3.6.3 package) (http://www.R-project.org/). We also used R (3.6.3) to construct Venn diagrams to show the unique and overlapping OTU between the animal groups. The Wilcoxon rank-sum test was used to detect the significant effects of acorn/tannin addition on the gut microbiota at both the phylum and genus levels between different groups of chipmunks or mice.

#### LEfSe analysis

We examined the dissimilarities among the animal groups by performing linear discriminant analysis effect size (LEfSe, v1.0) and the Kruskal–Wallis sum-rank test. Linear discriminant analysis (LDA) was then performed to determine the size effect of each distinctively abundant taxon from species to phylum.

#### Functional prediction of the microbial genes

We predicted the metabolic functional profile of the gut microbiota of chipmunks and mice using phylogenetic investigation of communities by reconstruction of unobserved states (PICRUSt) according to the Kyoto Encyclopedia of Genes and Genomes (KEGG) database. We used the obtained OTU data to generate BIOM files formatted as input for PICRUSt v1.1.09 with the make.biom script usable in Mothur. We then mapped the OTU abundances to Silva OTU IDs as input to infer the function of the gut microbiota [51].

### Untargeted metabolome of serum

#### Sample preparation

Serum samples frozen at − 80 °C were transferred on dry ice to Shanghai Lumineers for analysis. Samples stored at − 80 °C were removed and thawed at room temperature, after which 100 μL of each sample was transferred to a 1.5-mL EP tube. Then, 300 μL of the protein precipitant methanol–acetonitrile (V:V = 2:1, containing L-2-chlorophenylalanine, 2 μg/mL) was added, and the mixture was shaken for 1 min. The solution was extracted by sonication in an ice water bath for 10 min and allowed to stand at − 40 °C for 30 min. Then, the mixture was centrifuged for 10 min (13,000 rpm, 4 °C), and 200 μL of the supernatant was evaporated in an LC‒MS injection vial. After that, the solution was redissolved in 300 μL of methanol–water (V:V = 1:4) (vortexing for 30 s, sonication for 3 min) and allowed to stand at − 40 °C for 2 h [52]. Finally, the mixture was centrifuged for 10 min (13,000 rpm, 4 °C), 150 μL of supernatant was aspirated, and the mixture was filtered through a 0.22-μm organic phase pinhole filter, transferred to an LC injection vial, and stored at − 80 °C until LC‒MS analysis.

#### LC‒MS analysis

The analytical instrument for the experiments was a liquid mass spectrometry system consisting of an ACQUITY UPLC I-Class plus ultraperformance liquid tandem QE high-resolution mass spectrometer. An ACQUITY UPLC HSS T3 column (100 mm × 2.1 mm, 1.8 µm) was used. The column temperature was set at 45 °C. The mobile phase was composed of water (containing 0.1% formic acid) and acetonitrile (containing 0.1% formic acid). The flow rate was 0.35 mL/min, and the injection volume was 2 μL [53]. The raw data were processed using Progenesis QI v2.3 metabolomics software (Nonlinear Dynamics, Newcastle, UK) for baseline filtering, peak detection, identification and matching, retention time alignment, and normalization following the standard parameters: a precursor tolerance of 5 ppm/10 ppm and a product tolerance of 10 ppm/20 ppm [54].

#### Metabolite identification and analysis

The compounds were identified using the Human Metabolome Database (HMDB), LIPID MAPS (v2.3), and METLIN databases, complemented with a self-built library based on accurate mass numbers, secondary fragmentation, and isotopic distribution. Characterization was carried out. For the extracted data, the ion peaks with > 50% missing values (0 values) were removed from the group, and the 0 values were replaced by half of the minimum value. Therefore, we selected the compounds obtained from the characterization based on a score of the characterization results. We combined the positive and negative ion data into a data matrix table for subsequent analysis.

For the multivariate statistical analysis of the untargeted serum metabolome, we used unsupervised principal component analysis (PCA) to examine the general distribution between samples and whether the whole analysis process was stable. Then, we used partial least squares analysis (PLS-DA) and orthogonal partial least squares analysis (OPLS-DA) to distinguish general variations in metabolic profiles and detect different metabolites between the animal groups.

#### Measurement of short-chain fatty acids

The cecal samples were transported on dry ice to Shanghai Lumineers to analyze the short-chain fatty acids. According to the manufacturer’s instructions, the appropriate sample was weighed, and 300 μL of 50% acetonitrile–water solution (v/v) (containing the internal standard mix [2H9]-pentanoic acid, [2H11]-hexanoic acid, precooled at 4 °C) was added. The sample was ground for 3 min (precooled to − 20 °C) and sonicated in an ice-water bath for 10 min. Then, the sample was centrifuged at 4 °C and 2000 rpm for 10 min, after which 50 μL of the supernatant was diluted five times. For sample derivatization, 40 μL of 200 mM 3-NPH (50% aqueous acetonitrile configuration, v/v) and 40 μL of 120 mM EDC-6% pyridine (50% aqueous acetonitrile configuration, v/v) were added to the feed glass vial containing the extract, and the reaction was carried out at 40 °C for 30 min. Samples were cooled on ice for 1 min, and 200 μL of supernatant was aspirated, filtered through a 0.22-μm pinhole filter, transferred to an injection vial, and stored at − 80 °C until analysis. For the derivatization of standards, 80 μL of standard was added to a glass feed vial, and 40 μL of 200 mM 3-NPH (50% acetonitrile aqueous configuration, v/v) and 40 μL of 120 mM EDC-6% pyridine (50% acetonitrile aqueous configuration, v/v) were added and reacted at 40 °C for 30 min. We used UPLC-ESI–MS/MS to qualitatively and quantitatively detect the target metabolites in the cecal samples. The quantification of short-chain fatty acids in cecal samples was performed using triple quadrupole mass spectrometry in multireaction detection (MRM) mode. Identification and integration of each MRM transition were performed using the default parameters embedded in the SCIEX OS-MQ software (Sciex, USA).

### Hippocampal proteomics

#### Protein extraction and digestion

The hippocampal samples from the TST and TSC groups were transported by dry ice to Shanghai Jingjie Biological Company for proteomic analysis. The sample was ground with liquid nitrogen to a cell powder and transferred to a 5-mL centrifuge tube according to the manufacturer’s instructions. Then, four volumes of lysis buffer containing 8-M urea and 1% protease inhibitor cocktail were added to the cell powder, followed by sonication three times on ice using a high-intensity ultrasonic processor (Scientz). We removed the remaining debris by centrifugation for 10 min at 4 °C and 12,000 × g. The supernatant was collected for determination of the protein concentration using a BCA kit. To facilitate trypsin digestion, we first reduced the protein solution with 5-mM dithiothreitol at 56 °C for 30 min and then alkylated it with 11-mM iodoacetamide at room temperature in darkness for 15 min. Afterwards, the protein sample was diluted by adding 100-mM TEAB to urea to a concentration less than 2 M. For the first overnight digestion, we added trypsin at a 1:50 trypsin-protein mass ratio and changed it to a 1:100 trypsin-protein mass ratio for a second 4-h digestion [55]. Finally, we used a Strata X C18 SPE column (Phenomenex) to desalt the peptides before TMT labeling.

#### TMT labeling

Tryptic peptides were dissolved in 0.5-M TEAB, and each channel of the peptide was labeled with the respective TMT reagent (Thermo Fisher Scientific) and incubated for 2 h at room temperature according to the manufacturer’s protocol. Five microliters of each sample was pooled, desalted, and analyzed by MS to ensure labeling efficiency. The samples were quenched by adding 5% hydroxylamine [56].

#### HPLC fractionation

Following desaltination and desiccation by vacuum centrifugation, the sample was fractionated into 80 fractions using high-pH reversed-phase HPLC equipped with an Agilent 300 Extend C18 column (5-μm particles, 4.6-mm ID, 250-mm length) with a gradient of 2 to 60% acetonitrile (pH = 10) at 10-mM ammonium bicarbonate for 80 min. The peptides were then combined into nine fractions and dried by vacuum centrifugation for MS analysis [56].

#### LC‒MS/MS analysis

The peptides were separated by an ultrahigh performance liquid phase system, ionized into an NSI ion source, and then analyzed by Orbitrap Exploris™ 480 (Thermo Fisher Scientific) mass spectrometry. The column was eluted with a mobile phase comprising (A) 0.1% formic acid in 2% acetonitrile and (B) 0.1% formic acid in 90% acetonitrile. The liquid phase gradient conditions were 0–4 min (7–11% B), 4–53 min (11–32% B), 53–57 min (32–80% B), and 57–60 min (80% B) at a constant flow rate of 500 nL/min [57]. The ion source voltage was set at 2300 V, and the FAIMS compensation voltage (CV) was set at − 45 V. Full-scan primary mass spectrometry data were acquired in the range of 400–1200 m/z in a 60,000 solution. The scanning range of the secondary mass spectrometer was fixed at 110 m/z in a solution of 30,000. The data acquisition mode used a data-dependent scanning (DDA) procedure. To improve the effective utilization of the mass spectrum, automatic gain control (AGC) was used to avoid repeated scanning of the parent ions.

#### Protein identification and bioinformatics analysis

We annotated the identified proteins based on our previously assembled chromosome-level genome of *T. sibiricus* [58]. Only peptides unique to a given protein were used for relative quantification. We normalized the quantification for each sample using the average ratio of all the unique peptides. The fold change (FC) was calculated, and the significance of differences between the two groups was tested based on quantitative results. We used the two-tailed Fisher’s exact test to detect the differentially expressed proteins (DEPs) versus all identified proteins. A *P*-value < 0.05 was considered to indicate statistical significance. The identified proteins whose calculated fold changes (FCs) were less than 1/1.3 or greater than 1.3 were regarded as DEPs in our study. Gene Ontology (GO) annotation (e.g., cellular component, molecular function, and biological process domains) of the DEPs between the two groups was performed according to the GO database (http://geneontology.org/) [59]. The eukaryotic cluster of the orthologous group (KOG) database (https://www.ncbi.nlm.nih.gov/research/cog-project/) was used to analyze the functional classification of DEPs between the two chipmunk groups. Enrichment of the differentially expressed proteins between the two groups was analyzed using Fisher’s exact test for the functions GO, KEGG, protein domain, Reactome, and WikiPathways.

#### Data analysis

We used the Statistical Package for the Social Sciences (SPSS) v12.0 package (SPSS Inc., IL, USA) for the data analyses. We used the independent samples *t*-test to detect the difference in the proportion of seeds scatter-hoarded by the chipmunks after arc-sine transformation. The same procedure was applied for the differences in the concentrations of serum metabolites and short-chain fatty acids in the cecum. The performance of chipmunks in the eight-armed maze and mice in the Barnes maze was evaluated by using an independent samples *t*-test to identify significant differences, respectively. Spearman correlation was used to detect the connection of gut microbiota with the production of serum metabolites and short-chain fatty acids in the cecum , respectively.

## Results

### Effects of tannins on spatial memory and scatter hoarding in *T. sibiricus*

**Fig. 1:**
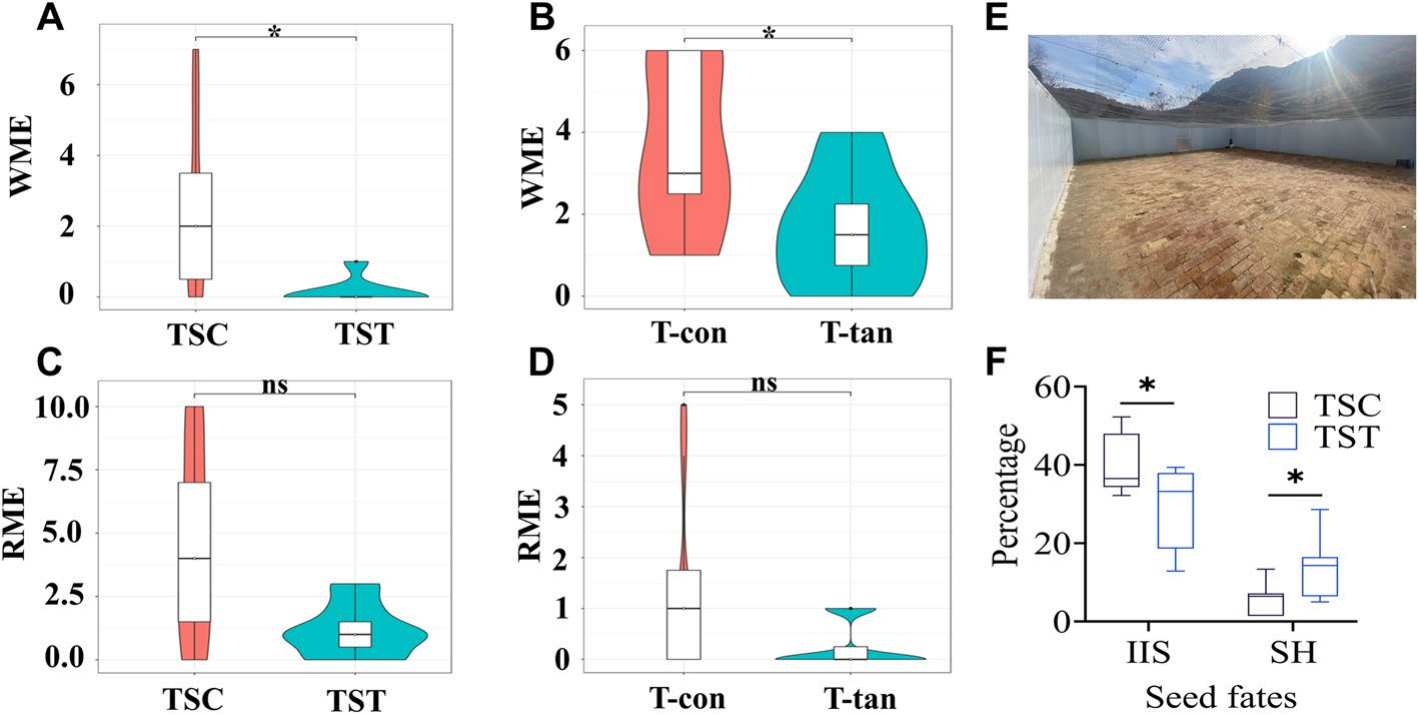
Effects of *Q. variabilis* high-tannin acorns and tannic acid on the spatial memory and hoarding behavior of *T. sibiricus*. **A** Effect of *Q. variabilis* acorns on the WME of *T. sibiricus* in the eight-armed maze. **B** Effect of tannic acid on the WME of *T. sibiricus* in the eight-armed maze. **C** Effect of *Q. variabilis* acorns on the RME of *T. sibiricus* in the eight-armed maze. **D** Effect of tannic acid on the RME of *T. sibiricus* in the eight-armed maze. **E** Artificial enclosures used in this study. **F** Box plots showing the percentage of seeds handled by *T. sibiricus* in the two groups; IIS, intact in situ; SH, scatter-hoarded. TSC and TST indicate the control and acorn treatment groups of chipmunks, respectively. T-con and T-tan indicate the control and tannin acid treatment groups of chipmunks, respectively. Note: *Represents statistical significance at *P* < 0.05 level

The addition of high-tannin acorns of *Q. variabilis* and tannic acid significantly decreased working memory errors (WMEs) in the eight-arm maze (*t* = 2.270, *df* = 12, *P* = 0.042; *t* = 2.213, *df* = 12, *P* = 0.044; Fig. 1A, B) but did not affect reference memory errors (*t* = 2.167, *df* = 14, *P* = 0.051; *t* = 1.784, *df* = 14, *P* = 0.096; Fig. 1C, D). Behavioral tests in the seminatural enclosures showed that chipmunks fed *Q. variabilis* acorns scatter-hoarded more seeds than did the control counterparts (*t* = 2.265, *df* = 12, *P* = 0.043), resulting in fewer intact seeds in situ (*t* = 2.27, *df* = 12, *P* = 0.042) (Fig. 1E).

### Effect of High-tannin acorns on the intestinal microbiota of *T. sibiricus*

**Fig. 2:**
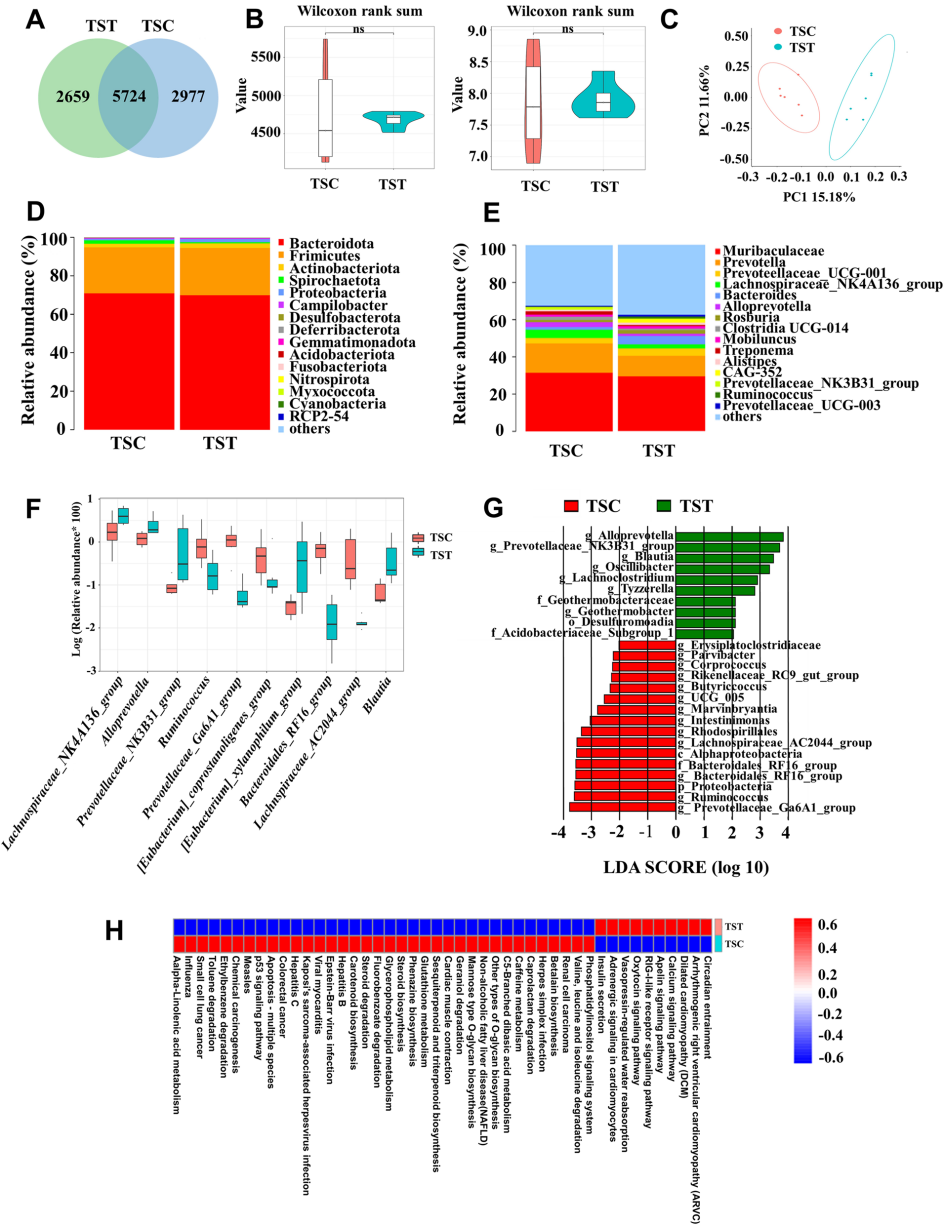
Effect of high-tannin acorns of *Q. variabilis* on the gut microbiota of *T. sibiricus*. **A** Venn diagram of OTU changes in the TST and TSC groups. **B** Chao1 and Shannon plots for the TST and TSC groups. **C** PCoA analysis of the intestinal microbiota in the TST and TSC groups. **D** Relative abundance of the gut microbiota at the phylum level in the TST and TSC groups. **E** Relative abundance of the gut microbiota at the genus level in the TST and TSC groups. **F** Box plot comparing the top 10 genera showing changes between the TST and TSC groups. **G** Linear discriminant analysis (LDA) effect size (LEfSe) analysis showing significant differences in the gut microbiota between the TST and TSC groups. The LDA score at the log10 scale is indicated at the bottom. The greater the LDA score is, the more significant the microbial biomarker is in the comparison. **H** Heatmap of the KEGG tertiary functional clustering in the TST and TSC groups. TSC and TST indicate the control and high-tannin acorn treatment groups of chipmunks, respectively

The rarefaction curves of the Chao1 and Shannon indices, rank abundance analysis curve, and specaccum species accumulation curve showed that the sequencing depth of all the fecal samples reached the saturation phase, indicating adequate sampling (Figure S1A, B). A total of 11,360 OTU were detected after removing the chimeric origin, with 2659 OTU specific to the TST group and 2977 OTU specific to the TSC group (Fig. 2A). The addition of high-tannin acorns significantly decreased the Chao1 and Shannon indices of the intestinal microbiota (Fig. 2B). In addition, β-diversity principal coordinate analysis of the diversity (PCoA) of the unweighted UniFrac (Anosim = 0.75, *P* = 0.003; Fig. 2C) and Bray‒Curtis (Anosim = 0.42, *P* = 0.004; Figure S2A) indices revealed significant differences in the intestinal flora between the TST and TSC groups of *T. sibiricus*. Analysis of the structural composition of the intestinal microbiota at the phylum level revealed that the dominant phyla for TST and TSC were *Bacteroides* (70.88% vs 69.99%, *P* = 0.73), Firmicutes (23.91% vs 24.55%, *P* = 0.71), Actinobacteria (1.87% vs 2.41% *P* = 0.26), Spirochaetota (1.78% vs 0.62%, *P* = 0.51), and Proteobacteria (0.71% vs 1.54%, *P* = 0.002) (Fig. 2D). The dominant genera in the TST and TSC groups were Muribaculaceae (31.46% vs 29.38%, *P* = 0.96), *Prevotella* (15.77% vs 11.00%, *P* = 0.52), *Prevotellaceae_UCG-001* (4.42% vs 2.17%,* P* = 0.17), *Lachnospiraceae_NK4A136_group* (3.18% vs 4.50%, *P* = 0.038), and *Bacteroides* (1.44% vs 4.46%, *P* = 0.19) (Fig. 2E). Compared to those in the TSC group, the top 10 most variable genera in the TST group were *Lachnospiraceae_NK4A136_*group (*P* = 0.038), *Alloprevotella* (*P* = 0.032), *Prevotellaceae_NK3B31_*group (*P* = 0.008), *[Eubacterium]_xylanophilum_*group (*P* = 0.044), and *Blautia* (*P* = 0.004), which were significantly increased in the TST group, and *Ruminococcus* (*P* = 0.015), *Prevotellaceae_Ga6A1_*group (*P* = 0.001), *[Eubacterium]_coprostanoligenes_*group (*P* = 0.037), *Bacteroidales_RF16_*group (*P* = 0.001), and *Lachnospiraceae_AC2044_*group (*P* = 0.001), which were significantly decreased in the TST group (Fig. 2F). Similarly, LEfSe analysis revealed that the abundances of *Alloprevotella*, *Prevotellaceae_NK3B31_*group, *Blautia*, and *Oscillibacter* were greater in the TST group, and the abundances of *Prevotellaceae_Ga6A1_*group, *Ruminococcus*, *Proteobacteria*, and *Bacteroidales_RF16_*group were greater in the TSC group (Fig. 2G). KEGG pathway enrichment analysis revealed that the calcium signaling pathway, the apelin signaling pathway, and the RIG-l-like receptor signaling pathway were significantly enriched in the TST group (Fig. 2H). We also investigated the effects of high-tannin acorns on the cecal microbiota of chipmunks, as indicated by the changes in alpha- and beta-diversities (Figure S3A, B, C, D), bacterial compositions at the genus and phylum levels (Figure S3E, F, G), and functions predicted by using KEGG (Figure S3H).

### Effect of tannic acid on the gut microbiota of *T. sibiricus*

**Fig. 3:**
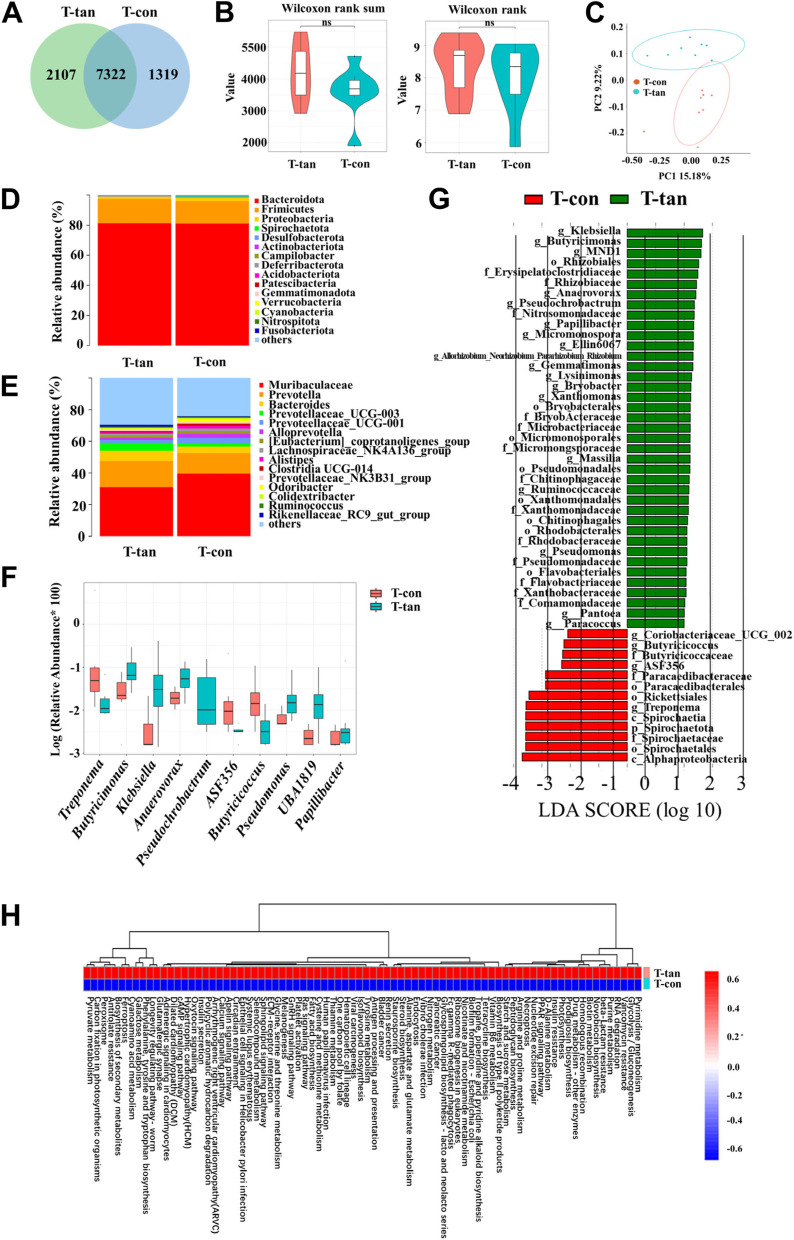
Effect of tannic acid on the gut microbiota of *T. sibiricus*. **A** Venn diagram of the OTU changes in the T-tan and T-con groups. **B** Chao1 and Shannon plots for the T-tan and T-con groups. **C** PCoA analysis of the gut microbiota in the T-tan and T-con groups. **D** Relative abundance at the phylum level in the gut microbiota of the T-tan and T-con groups. **E** Relative abundance of the intestinal microbiota at the genus level in the T-tan and T-con groups. **F** Box plot of comparative abundance between groups at the genus level for the T-tan and T-con groups. **G** LEfSe analysis of the gut microbiota in the T-tan and T-con groups. **H** Heatmap of the KEGG tertiary functional clustering of the T-tan group versus the T-con group. T-con and T-tan indicate the control and TA groups of chipmunks, respectively

The rarefaction curves of the Chao1 and Shannon indices, rank abundance analysis curve, and specaccum species accumulation curve indicated that the sequencing depth of all the samples reached the saturation phase, indicating adequate sampling (Figure S4A, B). After removing the chimeric origin, a total of 10,748 OTU were detected, with 1319 OTU specific to the T-con group and 2107 OTU specific to the T-tan group (Fig. 3A). The Chao1 and Shannon indices of the T-tan group were not significantly different from those of the T-con group (Fig. 3B). In addition, *β*-diversity principal coordinate analysis of diversity (PCoA) of the unweighted UniFrac distance (Anosim = 0.18, *P* = 0.024; Fig. 3C) and Bray‒Curtis distance (Anosim = 0.094, *P* = 0.094; Figure S2B) showed a significant separation between the T-con and T-tan groups. Analysis of the structural composition of the intestinal microbiota at the phylum level revealed that the dominant phyla of T-tan and T-con groups were Bacteroidota (81.23% vs 81.04%, *P* = 0.83), Firmicutes (16.27 vs 15.03%, *P* = 0.89), Proteobacteria (1.61% vs 2.18%, *P* = 0.14), Spirochaetota (0.02% vs 0.80%, *P* = 0.032), and Desulfobacterota (0.31% vs 0.43%, *P* = 0.6) (Fig. 3D). The dominant genera of T-tan and T-con were Muribaculaceae (31.02% vs 39.57%, *P* = 0.21), *Prevotella* (16.48% vs 12.85%, *P* = 0.24), *Bacteroides* (6.50% vs 3.84%, *P* = 0.33), *Prevotellaceae_UCG-003* (4.57% vs 4.24%, *P* = 0.31), and *Prevotellaceae_UCG-001* (1.93% vs 3.40%, *P* = 0.82) (Fig. 3E). At the genus level, *Butyricimonas* (*P* = 0.006), *Klebsiella* (*P* = 0.011), *Anaerovorax* (*P* = 0.003), *Pseudomonas* (*P* = 0.010), *UBA1819* (*P* = 0.038), Pseudochrobactrum (*P*=0.001), and *Papillibacter* (*P* = 0.040) increased significantly in the T-tan group, while *Treponema* (*P* = 0.034), *ASF356* (*P* = 0.036), and *Butyricicoccus* (*P* = 0.009) increased significantly in the T-con group (Fig. 3F). LEfSe analysis revealed a greater influence of *Klebsiella*, *Butyricimonas*, *MND1*, and Rhizobiales in the T-tan group and of Alphaproteobacteria, Spirochaetes, Spirochaetaceae, and Spirochaetota in the T-con group (Fig. 3G). KEGG pathway enrichment analysis revealed that the calcium signaling pathway, the apelin signaling pathway, and glycine, serine, and threonine metabolism were significantly enriched in the T-tan group (Fig. 3H).

### Effect of high-tannin acorns on the serum metabolites of *T. sibiricus*

**Fig. 4:**
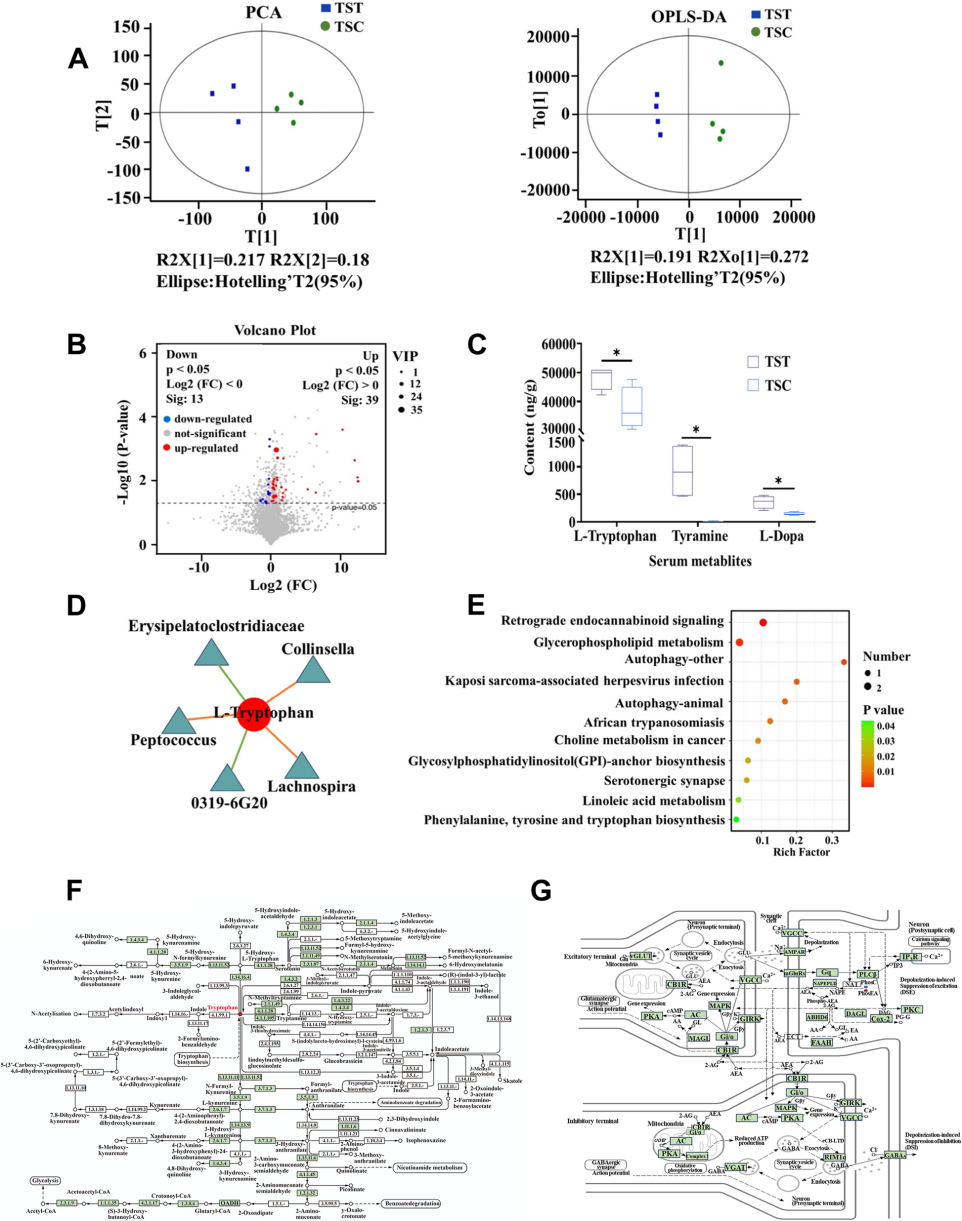
Effect of high-tannin acorns of *Q. variabilis* on serum metabolites of *T. sibiricus*. **A** PCA of the TST and TSC groups and OPLS-DA. **B** Volcano plot of differentially abundant metabolites between the TST and TSC groups. **C** Box plot of changes in serum metabolites in the TST group versus the TSC group. **D** Combined analysis showing the association of L-tryptophan with the gut microbiota. **E** Enrichment map of differential metabolic pathways in serum collected from the TST and TSC groups. The vertical coordinate is the name of the metabolic pathway; the horizontal coordinate is the Rich factor; the Rich factor = number of significantly different metabolites/number of total metabolites in the pathway; the higher the Rich factor is, the greater the enrichment. KEGG pathway showing tryptophan metabolism (**F**) and retrograde endocannabinoid signaling (**G**). TSC and TST indicate the control and acorn treatment groups of chipmunks, respectively

The PCA model has a cumulative R2X > 0.5, indicating the reliability and validity of the model. OPLS-DA showed that the sample distinction between the TST and TSC groups was significant at the 95% confidence interval, and that the R2Y values were close to 1, indicating that the model fit of the true picture of the sample (Fig. 4A). Thirteen serum metabolites were significantly downregulated, and 39 metabolites were significantly upregulated in the TST group compared to the TSC group (Fig. 4B). Among the neurotransmitters, L-tryptophan, tyramine, and L-DOPA were significantly more abundant in the TST group than in the TSC group (*t* = 2.575, *df* = 6, *P* = 0.042; *t* = 3.611, *df* = 6, *P* = 0.011; *t* = 3.576, *df* = 6, *P* = 0.012) (Fig. 4C). A combined analysis of the gut microbiota and the serum metabolome revealed correlations between the abundance of L-tryptophan and that of *Lachnospira*, *0319-6G20*, Erysipelatoclostridiaceae, *Peptococcus*, and *Collinsella* (Fig. 4D). KEGG enrichment analysis revealed that the metabolic pathways that differed significantly between the TST and TSC groups were retrograde endocannabinoid signaling, glycophospholipid metabolism, autophagy-other, Kaposi sarcoma-associated herpesvirus infection, African trypanosomiasis, choline metabolism in cancer, phenylalanine, tyrosine and tryptophan biosynthesis, serotonergic synapse, etc. (Fig. 4E, F, and G).

### Short-chain fatty acids in the cecal contents of chipmunks and mice

**Fig. 5:**
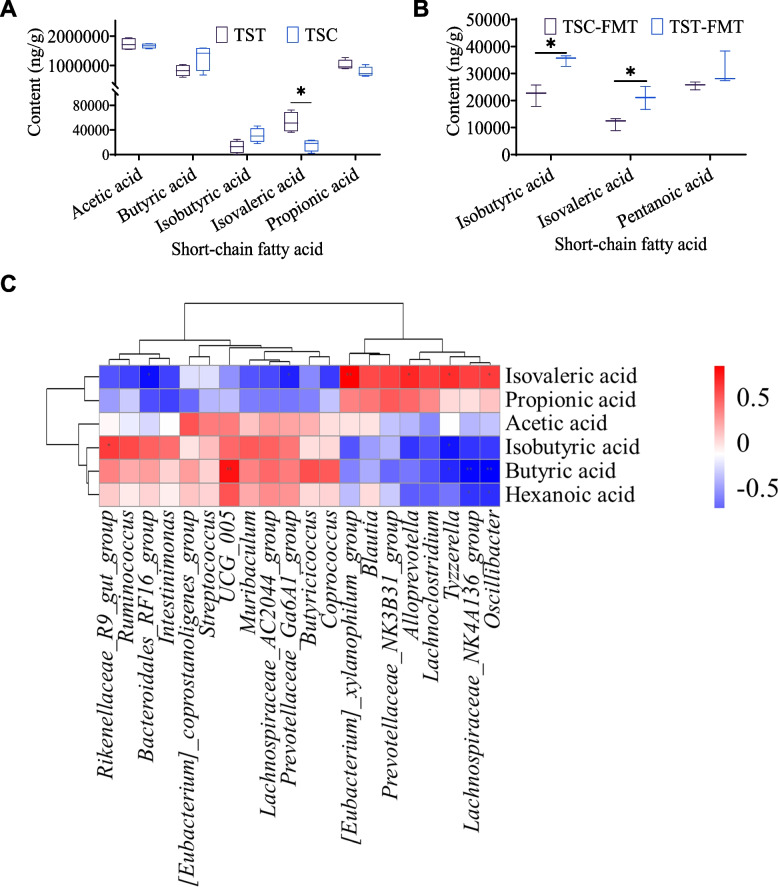
Effect of high-tannin acorns of *Q. variabilis* on short-chain fatty acids in *T. sibiricus*. **A** Short-chain fatty acid contents in the TST and TSC groups of *T. sibiricus*. **B** Short-chain fatty acid contents in the TST-FMT and TSC-FMT groups of mice. **C** Heatmap of the combined analysis of SCFAs and the gut microbiota in the TST and TSC groups. TSC and TST indicate the control and acorn treatment groups of chipmunks, respectively. Note: *Represents statistical significance at *P* < 0.05 level

The cecal concentrations of isovaleric acid (*t* = 4.008, *df* = 6, *P* = 0.007) in the chipmunks were significantly increased by the addition of high-tannin acorns (Fig. 5A). No significant changes were found in other short-chain fatty acids, such as acetic acid, butyric acid, pentanoic acid, or propionic acid (all *P* > 0.05). Moreover, mice in the TST-FMT group exhibited greater levels of isobutyric acid and isovaleric acid than did those in the TSC-FMT group (*t* = 4.957, *df* = 4, *P* = 0.008; *t* = 3.357, *df* = 4, *P* = 0.028) (Fig. 5B). The results of the combined analysis of short-chain fatty acids and the gut microbiota of chipmunks showed that isobutyric acid was only closely correlated with *Tyzzerella*, while isovaleric acid was closely correlated with spatial memory-related microbes such as *Oscillibacter*, *Tyzzerella*, *Alloprevotella*, *[Eubacterium]_xylanophilum_group*, *Prevotellaceae_Ga6A1_group*, and *Bacteroidales_RF16_group* (Fig. 5C).

### Effect of high-tannin acorns on the hippocampal proteome of *T. sibiricus*

In our study, we detected 29,232 peptides in the hippocampus of chipmunks, and 28,274 peptides covering 5682 quantifiable proteins were carefully identified with at least one unique peptide and an FDR confidence interval ≤ 0.01 (Figure S5A). The molecular weights of the 4642 identified proteins ranged from 5 to 100 kDa, while those of 1040 proteins exceeded 100 kDa (Figure S5B). We identified 163 proteins containing more than 20 peptides, while the remaining 5519 proteins had fewer than 20 peptides (Figure S5C). We also found that the proteins identified in our study had low proteome sequence coverage (Figure S5D), e.g., 1385 proteins had more than 20% sequence coverage. The majority of the detected proteins ranged from 2 to 3 charges and 7 to 20 amino acids in length (Figure S5E). The Pearson correlation coefficient between samples further verified that both trypsin digestion and LC–MS/MS detection were reliable and feasible in our study (Figure S5F).

**Fig. 6:**
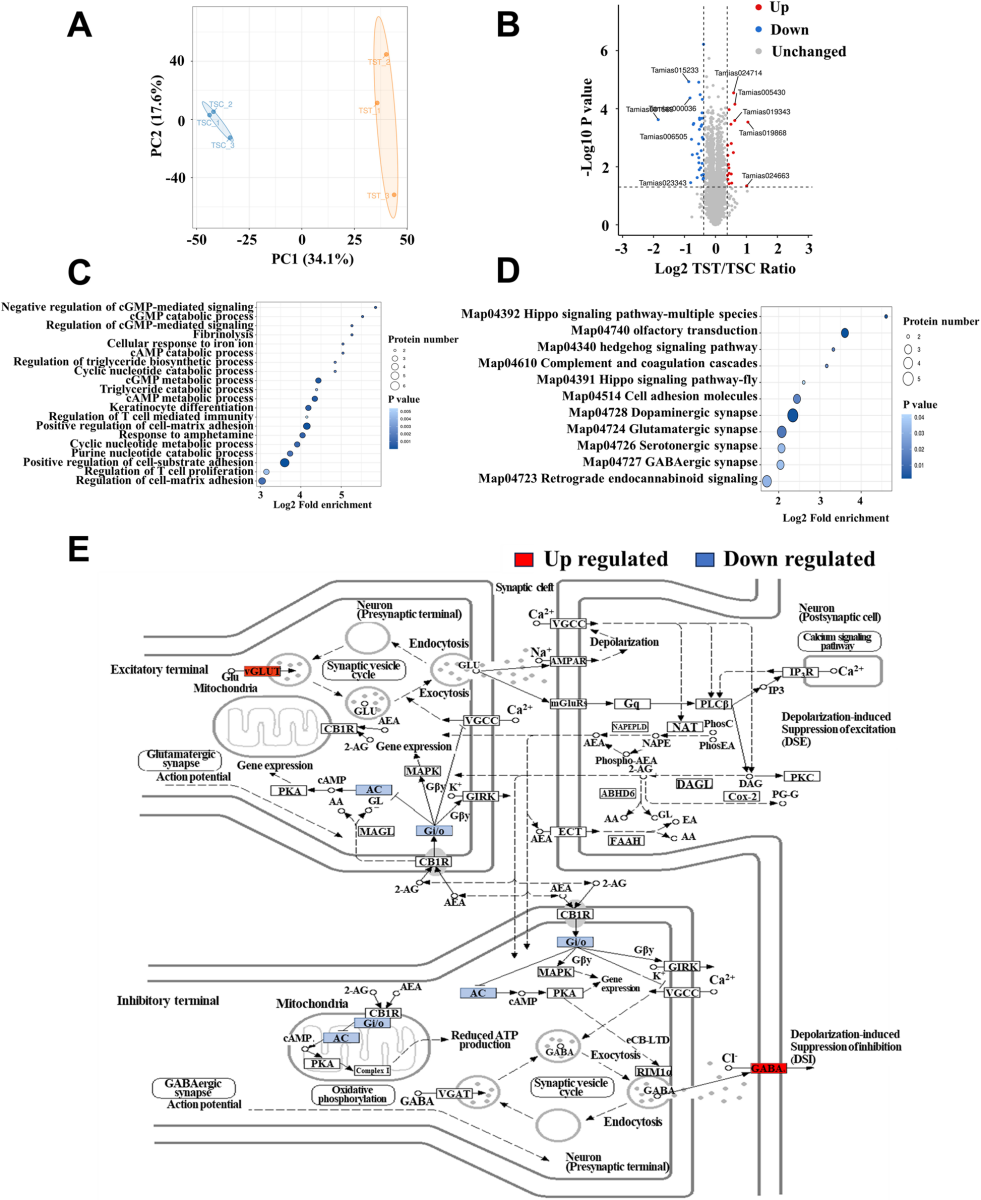
Effect of high-tannin acorns of *Q. variabilis* on the hippocampal proteome of *T. sibiricus*. **A** PCA plot of the hippocampal proteome of the TST versus TSC groups of *T. sibiricus*. **B** Volcano plot of differentially expressed proteins between the TST and TSC groups. **C** Bubble diagram of the enrichment of GO functions in the TST and TSC groups. The size of the blue dots indicates the number of differentially expressed proteins in the GO function, with larger dots indicating more differentially expressed proteins. **D** Bubble diagram of the KEGG enrichment in the TST and TSC groups. The size of the blue dots indicates the number of differential proteins in the KEGG pathway, and the larger dots indicate more differential proteins. **E** The retrograde endocannabinoid signaling pathway. TSC and TST indicate the control and acorn treatment groups of chipmunks, respectively

The PCA results showed that samples from the TST group were significantly separated from those from the TSC group (Fig. 6A). Compared with those in the TSC group, 19 proteins were significantly increased, and 35 proteins were significantly reduced in the TST group (*FC* > 1.3) (Fig. 6B). GO functional enrichment analysis revealed that the differentially expressed proteins were mainly enriched in signaling receptor binding, protein heterodimerization activity, enzyme inhibitor activity, cell adhesion molecule binding, amino acid transmembrane transporter activity, and other functions (Fig. 6C). KEGG pathway enrichment analysis revealed that the differentially expressed proteins were enriched mainly in retrograde endocannabinoid signaling, GABAergic synapses, serotonergic synapses, glutamatergic synapses, dopaminergic synapses, and other signaling pathways (Fig. 6D). Most notably, the protein expression of vesicular glutamate transporter 3 (VGLUT3) and the beta-1 gamma-aminobutyric acid receptor subunit (GABA_A_ R-beta1), which are involved in the regulation of the retrograde endocannabinoid signaling pathway, was significantly elevated (Fig. 6E).

### Effect of FMT on the spatial memory of C57BL/6 mice

**Fig. 7:**
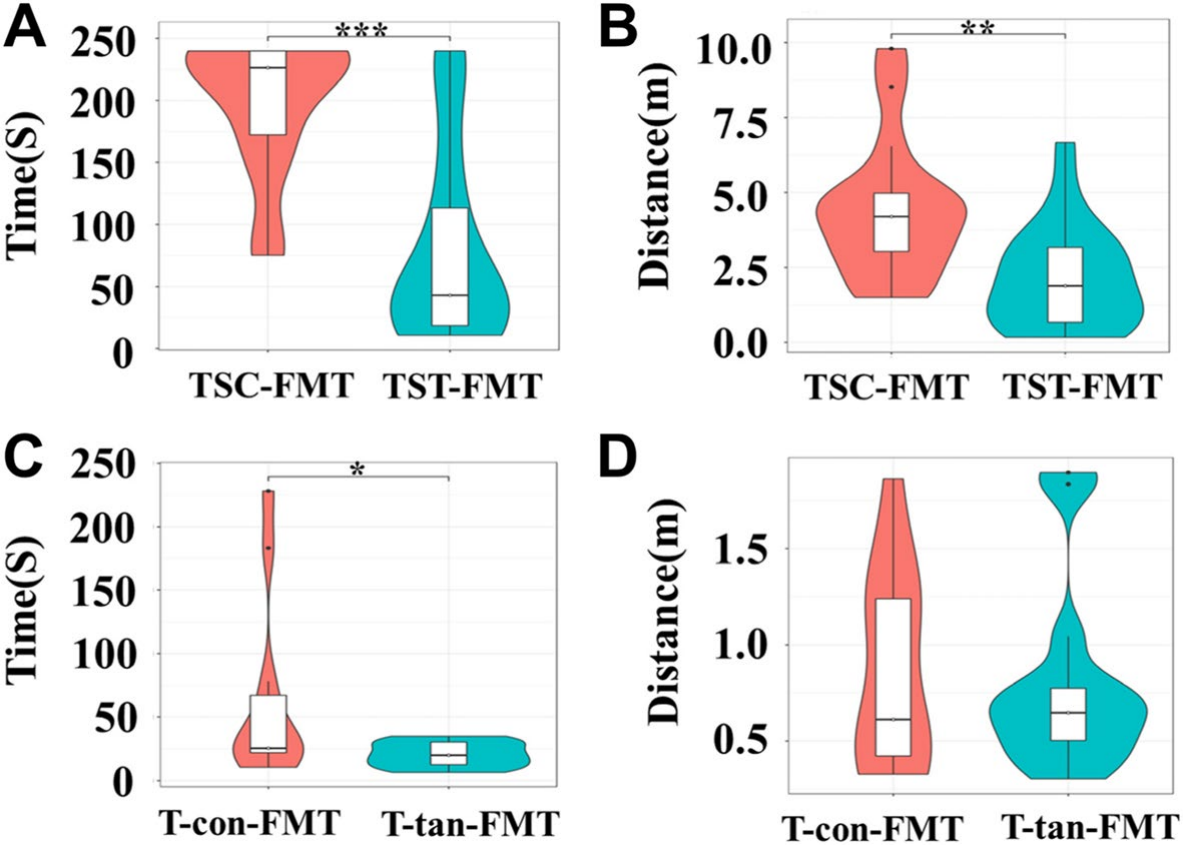
Effect of FMT on spatial memory in C57BL/6 mice. **A** Time to find the target hole in the TST-FMT and TSC-FMT groups. **B** Distance traveled to reach the target hole in the TST-FMT and TSC-FMT groups. **C** Time to reach the target hole in the T-tan-FMT and T-con-FMT groups. **D** Distance traveled to reach the target hole in the T-tan-FMT and T-con-FMT groups. For the TST-FMT and TSC-FMT groups, *N* = 18; for the T-tan-FMT and T-con-FMT groups, *N* = 12. The values are expressed as the means ± SDs. TST-FMT and TSC-FMT indicate mice that received fecal microbiota from the TSC and TST groups of chipmunks, respectively, while T-tan-FMT and T-con-FMT indicate mice that received fecal microbiota from the T-con and T-tan groups of chipmunks, respectively. Note: *, **, and ** represent statistical significance at *P* < 0.05, *P* < 0.01, and *P* < 0.001 levels, respectively

The results of the Barnes maze experiment showed that mice in the TST-FMT group searched for the target hole significantly faster than did those in the TSC-FMT group in terms of time spent and distance traveled (*t* = 5.167, *df* = 34, *P* < 0.001; *t* = 3.407, *df* = 34, *P* = 0.002) (Fig. 7A, B). Moreover, the T-tan-FMT group spent significantly less time locating the target hole than did the T-con-FMT group (*t* = 2.162, *df* = 22, *P* = 0.045) (Fig. 7C), while there were no significant differences between the two groups in terms of the distance traveled to locate the target hole (*t* = 0.2914, *df* = 22, *P* = 0.774) (Fig. 7D).

### Effect of FMT on the intestinal microbiota of C57BL/6 mice

**Fig. 8:**
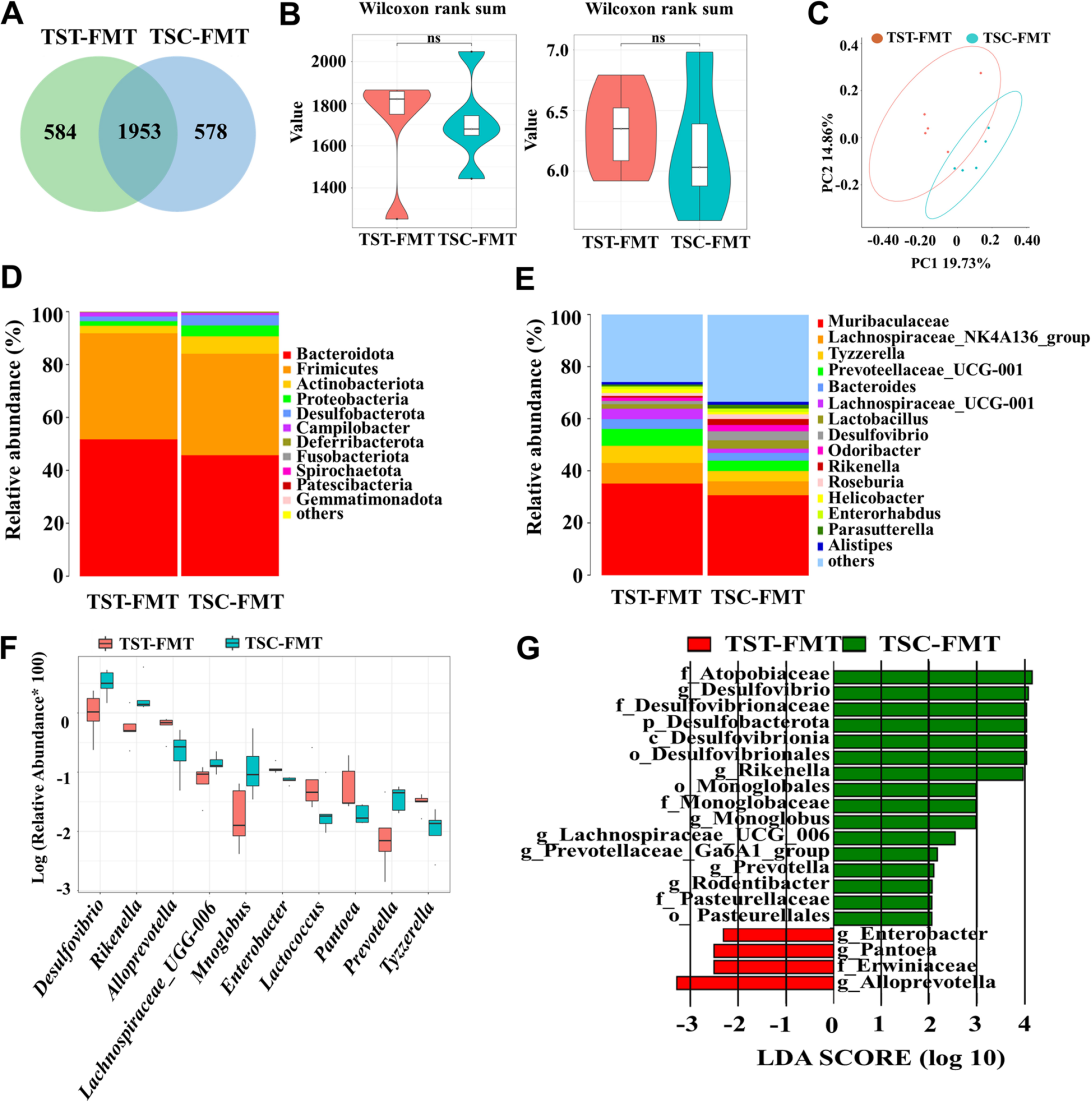
Changes in the gut microbiota of mice in the TST-FMT and TSC-FMT groups. **A** Venn diagram of the OTU changes in the TST-FMT and TSC-FMT groups. **B** Chao1 and Shannon plots for the TST-FMT and TSC-FMT groups. **C** PCoA of the intestinal microbiota in the TST-FMT and TSC-FMT groups. **D** Relative abundance of the gut microbiota at the phylum level in the TST-FMT and TSC-FMT groups. **E** Relative abundance of the gut microbiota at the genus level in the TST-FMT and TSC-FMT groups. **F** Box plot comparing the abundances at the genus level between the TST-FMT and TSC-FMT groups. **G** LEfSe analysis of the gut microbiota in the TST-FMT and TSC-FMT groups. TST-FMT and TSC-FMT indicate mice that received fecal microbiota from the TST and TSC groups of chipmunks, respectively

The rarefaction curves of the Chao1 and Shannon indices, rank abundance analysis curve, and specaccum species accumulation curve indicated that the sequencing depth of all the samples reached the saturation phase, indicating adequate sampling (Figure S6A, B, C, D). In total, 3115 OTU were detected in the two groups after removing the chimeric origin. A total of 584 OTU were specific to the TST-FMT group, and 578 OTU were specific to the TSC-FMT group, with 1953 OTU shared by the two groups (Fig. 8A). Neither the Chao1 nor Shannon indices were significantly different between the two groups (Fig. 8B). Additionally, the unweighted UniFrac (Anosim = 0.48, *P* = 0.017) (Fig. 8C) and Bray‒Curtis (Anosim = 0.5, *P* = 0.024; Figure S2C) differences in *β*-diversity principal coordinate analysis of diversity (PCoA) showed that there was a significant separation in the mouse gut microbiota. At the phylum level, the dominant phyla in the TST-FMT and TSC-FMT groups were *Bacteroides* (51.73% vs 45.65%, *P* = 0.44), Firmicutes (40.10% vs 38.36%, *P* = 0.73), Actinobacteria (2.81% vs 6.58%, *P* = 0.06), Proteobacteria (1.72% vs 4.12%, *P* = 0.23), and Desulfobacterota (1.81% vs 3.90%, *P* = 0.021) (Fig. 8D). The dominant genera for TST-FMT and TSC-FMT were Muribaculaceae (35.15% vs. 30.79%, *P* = 0.44), *Lachnospiraceae_NK4A136_group* (7.86% vs. 5.31%, *P* = 0.73), *Tyzzerella* (6.60%, 3.97%, *P* = 0.31), *Prevotellaceae_UCG-001* (6.49% vs 3.97%, *P* = 0.26), and *Bacteroides* (3.76% vs 2.98%, *P* = 0.12) (Fig. 8E). Compared to those in the TSC-FMT group, the 10 most variable genera in the TST-FMT group were *Desulfovibrio* (*P* = 0.021), *Rikenella* (*P* = 0.033), *Alloprevotella* (*P* = 0.018), *Lachnospiraceae_UCG-006* (*P* = 0.027), *Monoglobus* (*P* = 0.035), *Enterobacter* (*P* = 0.001), *Lactococcus* (*P* = 0.047), *Pantoea* (*P* = 0.014), *Prevotella* (*P* = 0.03), and *Tyzzerella* (*P* = 0.006) (Fig. 8F). The results of the LEfSe analysis showed a prominence of *Alloprevotella*, Erwiniaceae, and *Pantoea* in the TST-FMT group but of Atopobiaceae, Desulfovibrionaceae, and Desulfobacterota in the TSC-FMT group (Fig. 8G).

## Discussion

In this study, we clearly showed that the addition of high-tannin acorns of *Q. variabilis* significantly improved the spatial memory of the scatter-hoarding rodent *T. sibiricus*, as indicated by its performance in the eight-arm maze. Tannic acid alone had a similar effect on the spatial cognition of *T. sibiricus*, further strengthening the key role of seed tannins in altering the spatial cognition of acorn-eating animals. Although the high-tannin hypothesis posits that food hoarding animals prefer to cache high-tannin foods, the underlying behavioral mechanism remains unclear [45]. Our study may provide reliable evidence that bitter tannins act as hidden players to benefit food-hoarding animals despite their toxicity to body weight, digestion, and absorption [10, 11]. Previous studies have shown that tannins or tannin-related extracts can inhibit nerve cell death in the brain or improve the spatial memory of animals and humans [60, 61], further supporting our speculation that tannins may act as a double-edged sword in the interactive relationship between plants and animals. Additionally, seed tannins significantly regulate the food-hoarding behavior of *T. sibiricus*, supporting the findings of a previous study showing that rodents with better spatial memory ability tend to cache more seeds [62]. Although tannins have long been considered one of the most important secondary metabolites by which plants regulate the food-hoarding behavior of rodents [45, 63], both the improved spatial memory and enhanced food-hoarding behavior of *T. sibiricus* in our study may explain why animals prefer high-tannin foods during food-hoarding processes. Although germ-free mice were not used in our study, a previous study has shown that the memory ability of the antibiotic depletion mice administrated by gavage of Triphala (tannin containing) is greatly improved [64]. Therefore, it is safe to anticipate that administrating tannins to antibiotic depletion mice will generate robust results. A recent study showing that the profile of the gut microbiota is closely related to the scatter-hoarding behavior of *Apodemus draco* [32] provides further support for our prediction that tannins enhance hoarding intensity via modification of the gut microbiota of *T. sibiricus*. Moreover, improved spatial memory by tannins will allow chipmunks to gain more food from their caches because they mainly rely on their memory and olfaction to selectively pilfer seeds from caches of other animals [7]. Therefore, plants are expected to benefit from manipulating spatial memory ability and the hoarding behavior of seed-eating rodents by producing tannins to carry out effective seed dispersal and natural regeneration.

Along with the improvement in spatial memory, both high-tannin acorns and tannic acid significantly altered the intestinal microbiota of *T. sibiricus*, as indicated by changes in *β*-diversity rather than *α*-diversity. Changes in the gut microbiota are consistent between acorn and tannic acid treatments, reflecting the crucial role of polyphenols in influencing the abundance and structure of the gut flora of animals [65]. The increase in Firmicutes but the decrease in *Bacteroides* under the treatments of acorns and tannic acid reflect the typical influence of polyphenols on the gut microbiota [66]. Specifically, genera such as *Lachnospiraceae_NK4A136*, *Prevotellaceae_NK3B31_group*, *Alloprevotella*, and *Blautia* were significantly upregulated by the addition of high-tannin acorns. The butyrate-producing *Lachnospiraceae_NK4A136* plays an important role in neuroprotection, cognitive improvement, and antidepressant effects [67, 68]. The decrease in *Prevotellaceae_NK3B31_group* and *Blautia* in the gut microbiota has been found to be closely related to cognitive impairment or a decline in spatial memory in mice [69–71]. In addition, the abundance of *Alloprevotella* has been shown to increase in mice with improved cognitive function [72, 73]. Furthermore, the abundance of *Butyricimonas*, closely related to the production of butyrate, which can improve spatial memory [74], increased in the gut of *T. sibiricus* after the addition of tannic acid. However, the abundance of *Butyricicoccus*, which is positively correlated with cognitive impairment [75], decreased significantly in response to tannic acid. Moreover, bacterial genera *Blautia*, *Klebsiella*, and *Pseudomonas* detected in the chipmunks either consuming acorns or tannic acid were found to participate in the methionine pathway, tryptophan-derived pathway, glutamate-derived pathway, and acetate pathway according to the gut-brain and gut-metabolic modules [76, 77]. In addition, these genera play a crucial role in lipolytic, proteolytic, and saccharolytic metabolism [76, 77], further pointing to similar effects of acorns and tannic acids on the gut microbiota of *T. sibiricus*. Therefore, changes in the gut microbiota related to cognition are expected to be highly responsible for improving the spatial memory of chipmunks in response to the addition of seed tannins. Spatial memory was improved in mice received bacterial suspension from donors either consuming acorns or tannic acid, reinforcing our view that the gut microbiota pathway acts as the main pathway by which seed tannins improve spatial memory of scatter-hoarding animals. We admit that *Quercus* acorns contain flavonoids and other polyphenols besides tannins [78], which have been shown to alter the gut microbiota and improve spatial memory or cognitive function [79–82]. Apart from the effects of tannins on gut microbiota, intestinal bacteria play an important role in decomposing tannins [83, 84], to produce short-chain fatty acid production [85, 86]. Therefore, gut microbiota may interact with seed tannins in regulating spatial cognition of hoarding animals.

L-Tryptophan, an essential amino acid and an important metabolic substrate for the synthesis of 5-HT through the 5-hydroxytryptamine pathway, acts as an important neurotransmitter in the regulation of spatial memory. Serum tryptophan can cross the blood–brain barrier and further increase the amount of 5-HT in the brain [87]. Reduced 5-HT levels in the brain lead to impaired learning and spatial memory in rats and mice [88, 89]. In contrast, increased levels of 5-HT in the brain can improve memory and cognitive function [90, 91]. Our study showed that the addition of high-tannin acorns significantly increased the serum level of L-tryptophan in *T. sibiricus*. The combined analysis of serum metabolomes and the gut microbiota showed a close correlation between the abundance of L-tryptophan and *Lachnospira* and other genera, suggesting that the increase in L-tryptophan mediated by the gut microbiota appears to be one of the molecular mechanisms by which seed tannins improve the spatial memory of *T. sibiricus*.

Like neurotransmitters, short-chain fatty acids synthesized by intestinal bacteria are also important for the regulation of brain function [25, 26]. Most importantly, changes in the gut microbiota of chipmunks and mice modulated by the addition of tannins are closely related to the production of isovaleric acid and isobutyric acid, which have been hypothesized to act as substrates to provide an alternative energy source to rectify neuronal dysfunction [92–94]. However, a reduction in isovaleric acid and isobutyric acid in the cecum has been accompanied by synaptic loss and memory impairment in mice [95, 96]. The combined analysis showed that the upregulation of isovaleric acid in the intestinal tract of *T. sibiricus* induced by the addition of acorns is closely associated with spatial memory-mediated microbes such as *Alloprevotella* and *Tyzzerella*, highlighting the vast possibility that the microbiota-gut-brain axis regulates the spatial memory of food-hoarding rodents through interactions between seed tannins and the intestinal microbiota.

It is widely accepted that spatial memories are highly dependent on the integrity of the hippocampus of animals. Therefore, changes in hippocampal structure and function will lead to modifications in the spatial memory of animals [97, 98]. In this study, we provide a detailed account of the hippocampal proteins of *T. sibiricus* using a tandem mass tag (TMT). Hippocampal proteome analyses revealed that vesicular glutamate transporter 3 (VGLUT3), which is closely related to cognitive ability, was upregulated by the addition of high-tannin acorns. Evidence has shown that increased VGLUT3 can reduce cognitive deficits in rats [99], while VGLUT3 knockout mice exhibit impaired memory and reduced cognitive flexibility [100], possibly because of a metaplastic change in synaptic plasticity in CA1 synapses due to loss of VGLUT3 [101]. Additionally, VGLUT3 is capable of promoting the vesicular accumulation and transmission of 5-HT and endows 5-HT fibers with the ability to use glutamate in fast excitatory neurotransmission [102, 103]. Therefore, it can be anticipated that the tannin-induced accumulation of VGLUT3 in the hippocampus contributes to improving the spatial memory of *T. sibiricus*. Furthermore, the beta-1 gamma-aminobutyric acid receptor subunit (GABA_A_ R-beta1) protein, a receptor for gamma-aminobutyric acid (GABA) that plays a central role in the regulation of cortical excitability and maintaining excitatory/inhibitory balance, was also upregulated by high-tannin acorns. Reduced GABA_A_ R-β1 expression is highly responsible for cognitive impairment in both Alzheimer’s disease patients [104] and mice [105]. KEGG pathway enrichment analysis revealed that VGLUT3 affects GABA_A_ R-β1 through the retrograde endocannabinoid signaling pathway, which is closely related to spatial memory [106]. Given that VGLUT3 has the potential to enhance vesicular GABA filling and promote glutamate release, we predict that elevated hippocampal GABA_A_ R-β1 expression through the retrograde endocannabinoid signaling pathway mediated by elevated expression of VGLUT3 may be one of the mechanisms by which seed tannins improve the hippocampal function of *T. sibiricus*.

Overall, in this study, we elucidated the effects of seed tannins on the spatial memory and food hoarding behavior of a scatterhoarding rodent in a multidimensional manner from plant secondary metabolites to gut microbes, hippocampal function, and spatial cognition. Our results demonstrated that seed tannins improve hippocampal function and ultimately modulate spatial memory through the gut microbiota-brain axis pathway. Gut microbiota-mediated changes in serum neurotransmitters such as L-tryptophan, as well as short-chain fatty acids such as isovaleric acid and isobutyric acid, act as crucial molecular mechanisms by which seed tannins improve spatial memory in *T. sibiricus*. The upregulation of VGLUT3 and GABA_A_ R-beta1 in the hippocampus by the addition of high-tannin acorns may also play a vital role in improving the spatial memory of *T. sibiricus*. Given the mutually beneficial coevolutionary relationships between food-hoarding rodents and plant seeds, our findings may have resolved the longstanding puzzle about the hidden role of plant secondary metabolites in manipulating food-hoarding behavior of rodents via the microbiota-gut-brain axis, which is of great importance for better understanding the mutualistic coevolution between plants and animals.

### Supplementary Information

Supplementary file 1: Figure S1: Gut microbiota analysis of chipmunks from the TST and TSC groups. Alpha rarefaction plot of Chao 1 and Shannon (A, B); Rank Abundance analysis curve (C); and Specaccum species accumulation curve (D). Figure S2: PCoA based on the Bray–Curtis distance matrix of the gut microbiota of chipmunks and mice. Chipmunks from the TSC and TST groups (A); chipmunks from the T-con and T-tan groups (B); and mice from TSC-FMT and TST-FMT groups (C). Figure S3: Effects of acorn tannins on the composition and function of cecal microbiota of chipmunks. Chao 1 and Shannon indices (A, B); Beta-diversity (PCoA) of the unweighted Unifrac distance and Bray–Curtis distance (C, D); Microbiota compositions at the phylum and genus levels (E, F); The linear discriminant analysis (LDA) effect size (LEfSe) analysis showing the significant difference of the cecal microbiota (G), and KEGG annotation of cecal microbiota (H). Figure S4: Gut microbiota analysis of chipmunks from the T-con and T-tan groups. Alpha rarefaction plot of Chao 1 and Shannon (A, B); Rank Abundance analysis curve (C); and Specaccum species accumulation curve (D). Figure S5: Pep-Quant library characteristics of chipmunks’ hippocampus. Bar graph showing distribution of (A) identified peptides and proteins, (B) molecular weight of proteins, (C) protein length, (D) proteome sequence coverage, (E) peptide charge, and (F) Pearson correlation coefficient. Figure S6: Gut microbiota analysis of mice from the TSC-FMT and TST-FMT group. Alpha rarefaction plot of Chao 1 and Shannon (A, B). Rank Abundance analysis curve (C); and Specaccum species accumulation curve (D).

## Data Availability

The 16S rRNA gene sequencing raw reads generated in this study were deposited into Figshare and can be downloaded freely at 10.6084/m9.figshare.24572665.
