# Supplementary material for: High-tannin food enhances spatial memory and scatter-hoarding in rodents via the microbiota-gut-brain axis
Source: Microbiome. 2024 Jul 29;12:140. doi: 10.1186/s40168-024-01849-2 (PMC11285206; doi:10.1186/s40168-024-01849-2)
Supplement: Supplementary file 2 — Supplementary file 1: Figure S1: Gut microbiota analysis of chipmunks from the TST and TSC groups. Alpha rarefaction plot of Chao 1 and Shannon (A, B); Rank Abundance analysis curve (C); and Specaccum species accumulation curve (D). Figure S2: PCoA based on the Bray–Curtis distance matrix of the gut microbiota of chipmunks and mice. Chipmunks from the TSC and TST groups (A); chipmunks from the T-con and T-tan groups (B); and mice from TSC-FMT and TST-FMT groups (C). Figure S3: Effects of acorn tannins on the composition and function of cecal microbiota of chipmunks. Chao 1 and Shannon indices (A, B); Beta-diversity (PCoA) of the unweighted Unifrac distance and Bray–Curtis distance (C, D); Microbiota compositions at the phylum and genus levels (E, F); The linear discriminant analysis (LDA) effect size (LEfSe) analysis showing the significant difference of the cecal microbiota (G), and KEGG annotation of cecal microbiota (H). Figure S4: Gut microbiota analysis of chipmunks from the T-con and T-tan groups. Alpha rarefaction plot of Chao 1 and Shannon (A, B); Rank Abundance analysis curve (C); and Specaccum species accumulation curve (D). Figure S5: Pep-Quant library characteristics of chipmunks’ hippocampus. Bar graph showing distribution of (A) identified peptides and proteins, (B) molecular weight of proteins, (C) protein length, (D) proteome sequence coverage, (E) peptide charge, and (F) Pearson correlation coefficient. Figure S6: Gut microbiota analysis of mice from the TSC-FMT and TST-FMT group. Alpha rarefaction plot of Chao 1 and Shannon (A, B). Rank Abundance analysis curve (C); and Specaccum species accumulation curve (D). [file 40168_2024_1849_MOESM1_ESM.docx]

**Figure S1**

Gut microbiota analysis of chipmunks from the TST and TSC groups. Alpha rarefaction plot of Chao 1 and Shannon (A, B); Rank Abundance analysis curve (C); and Specaccum species accumulation curve (D).


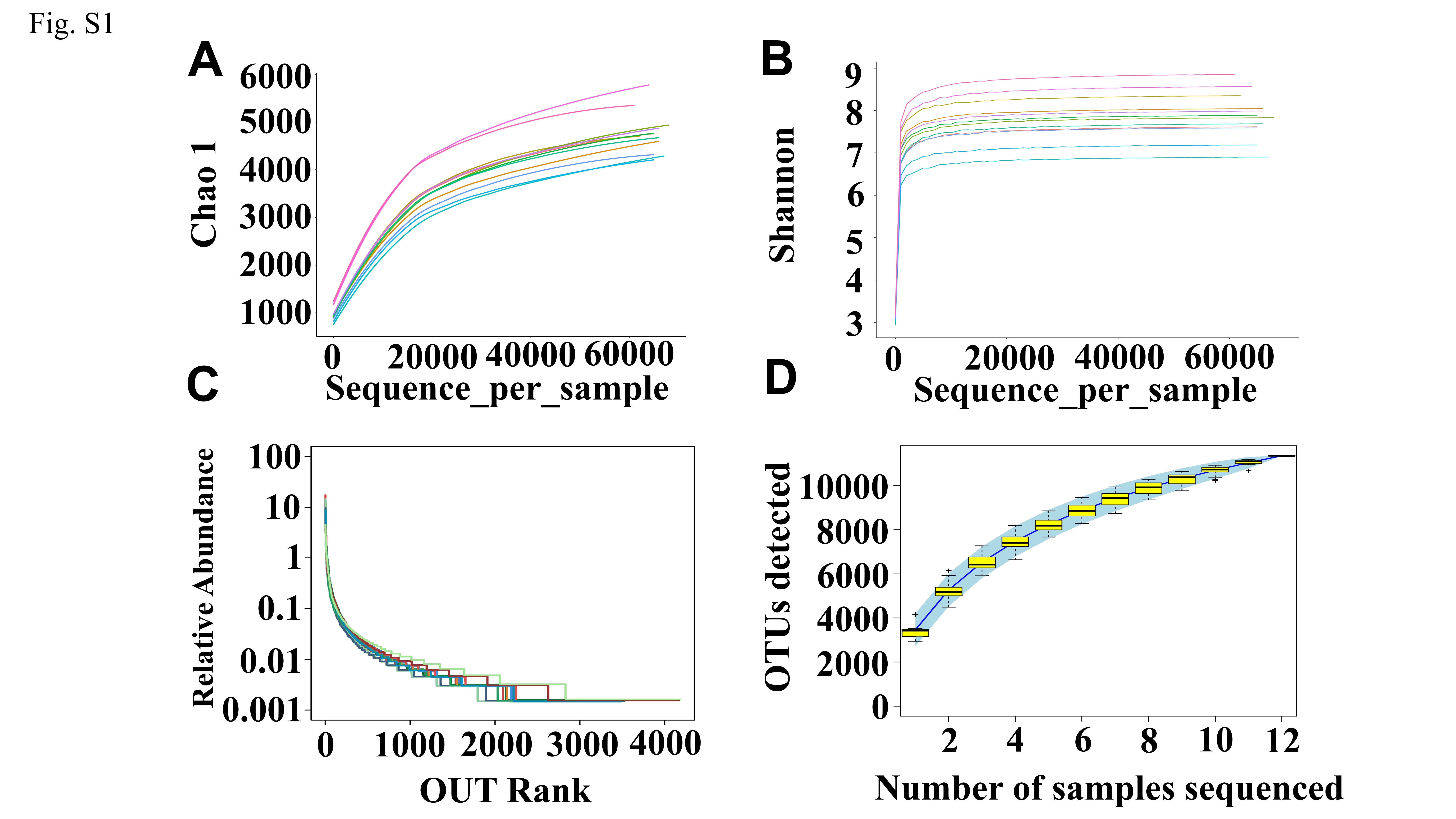


**Figure S2**

PCoA based on the Bray–Curtis distance matrix of the gut microbiota of chipmunks and mice. Chipmunks from the TSC and TST groups (A); chipmunks from the T-con and T-tan groups (B); and mice from TSC-FMT and TST-FMT groups (C).

**
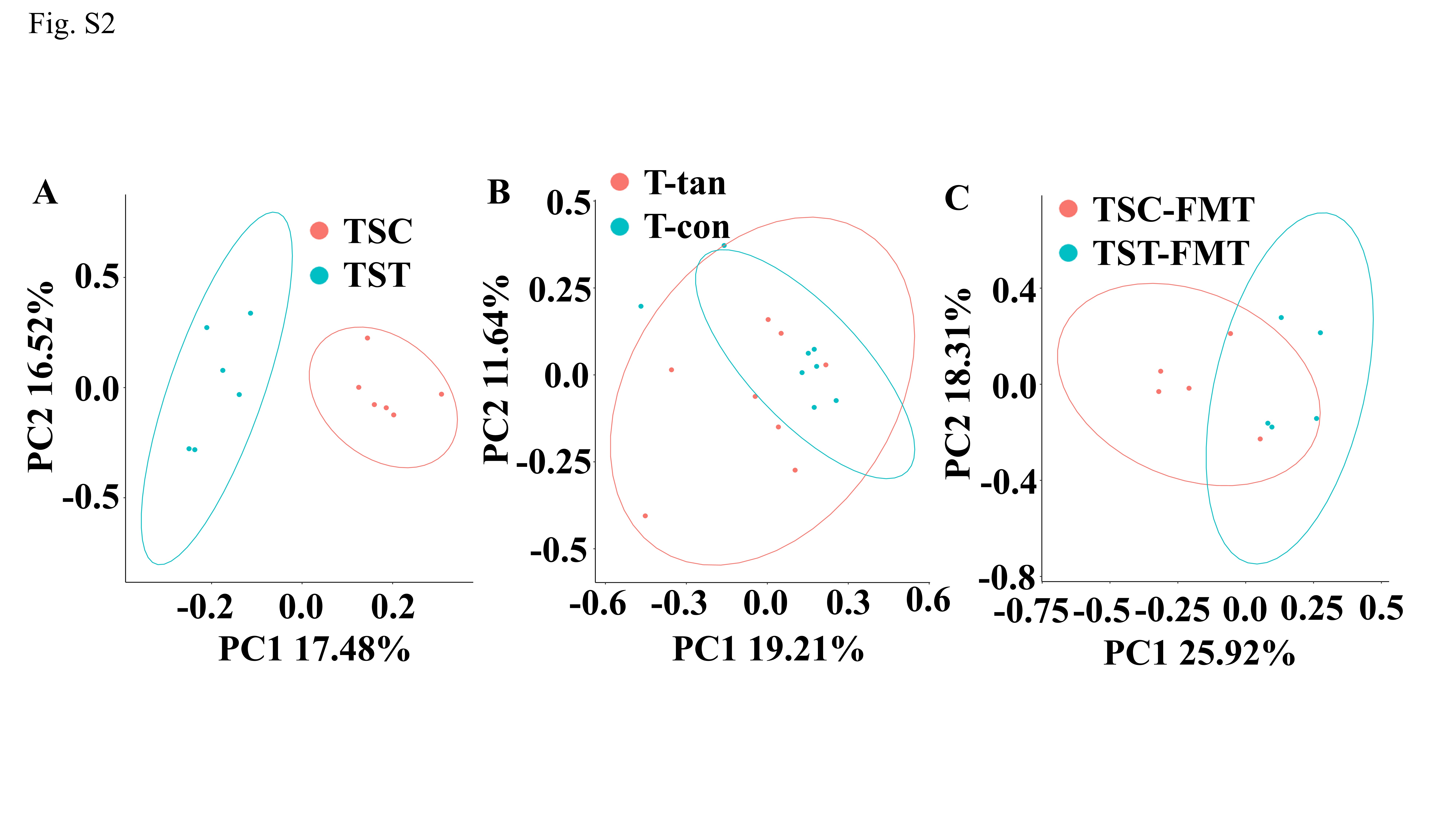
**

**Figure S3**

Effects of acorn tannins on the composition and function of cecal microbiota of chipmunks. Chao 1 and Shannon indices (A, B); Beta-diversity (PCoA) of the unweighted Unifrac distance and Bray-Curtis distance (C, D); Microbiota compositions at the phylum and genus levels (E, F); The linear discriminant analysis (LDA) effect size (LEfSe) analysis showing the significant difference of the cecal microbiota (G), and KEGG annotation of cecal microbiota (H).


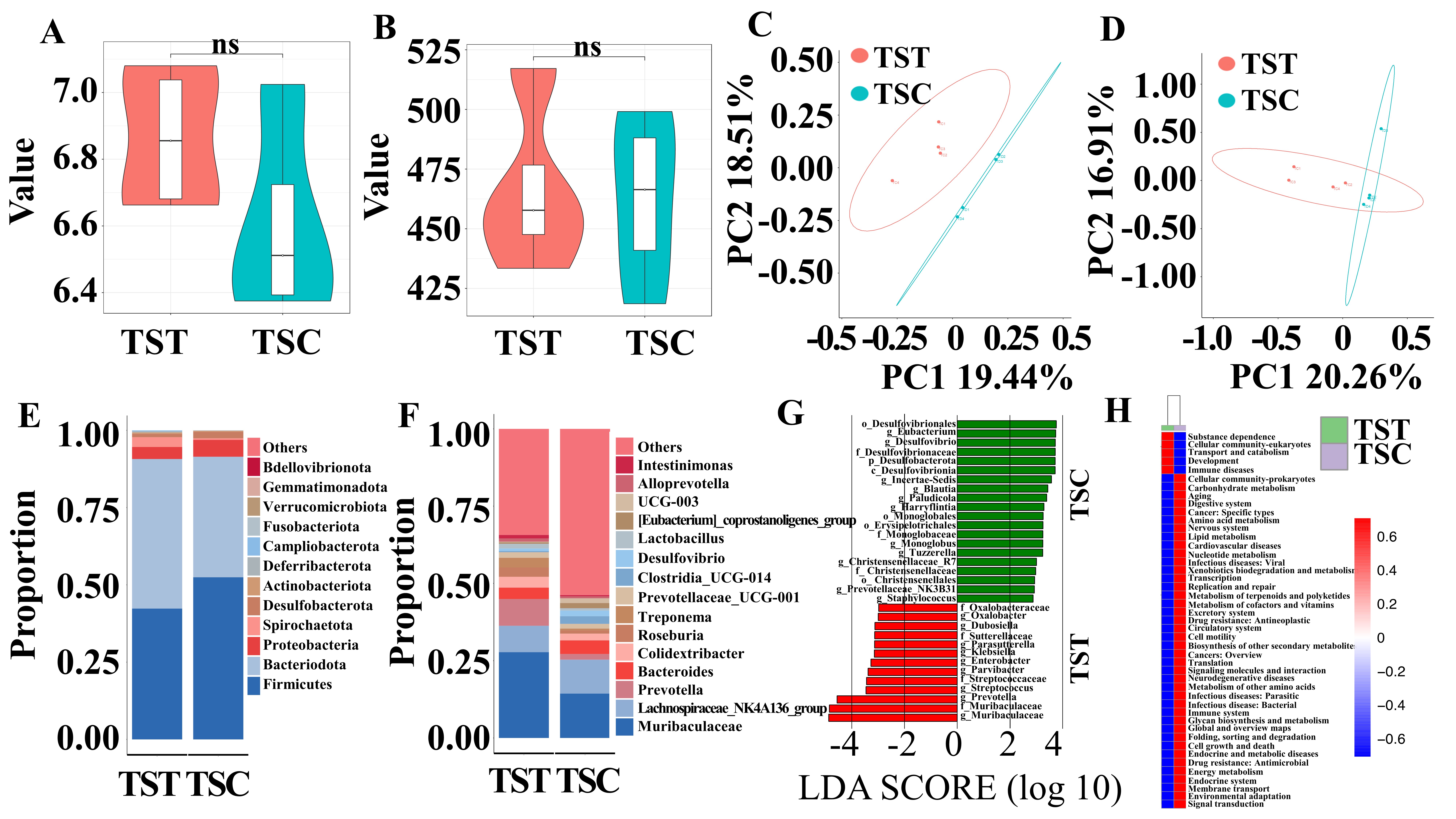


**Figure S4**

Gut microbiota analysis of chipmunks from the T-con and T-tan groups. Alpha rarefaction plot of Chao 1 and Shannon (A, B); Rank Abundance analysis curve (C); and Specaccum species accumulation curve (D).

**
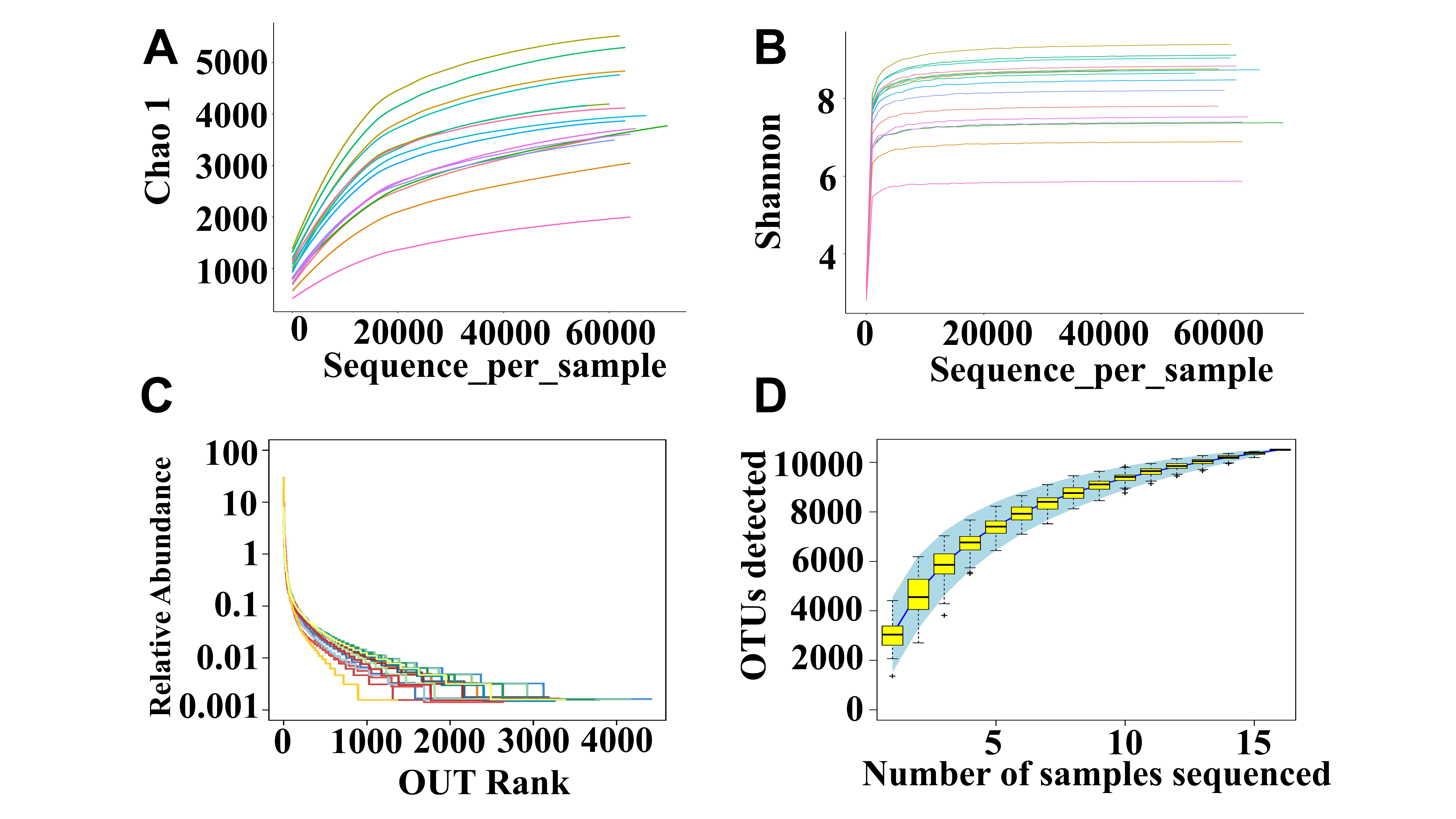
**

**Figure S5**

Pep-Quant library characteristics of chipmunks’ hippocampus. Bar graph showing distribution of (A) identified peptides and proteins, (B) molecular weight of proteins, (C) protein length, (D) proteome sequence coverage, (E) peptide charge, and (F) Pearson correlation coefficient.


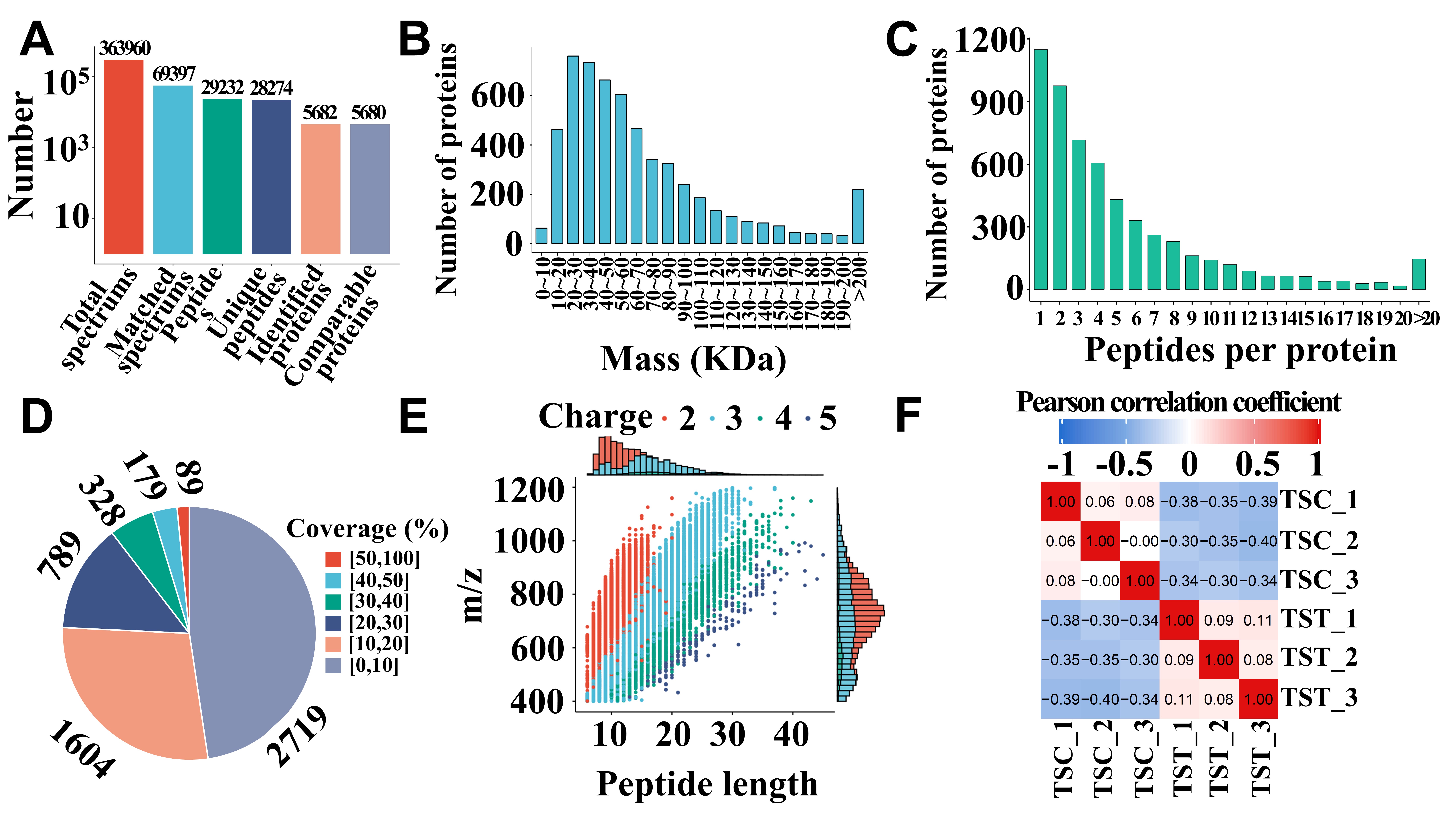


**Figure S6**

Gut microbiota analysis of mice from the TSC-FMT and TST-FMT group. Alpha rarefaction plot of Chao 1 and Shannon (A, B). Rank Abundance analysis curve (C); and Specaccum species accumulation curve (D).

**
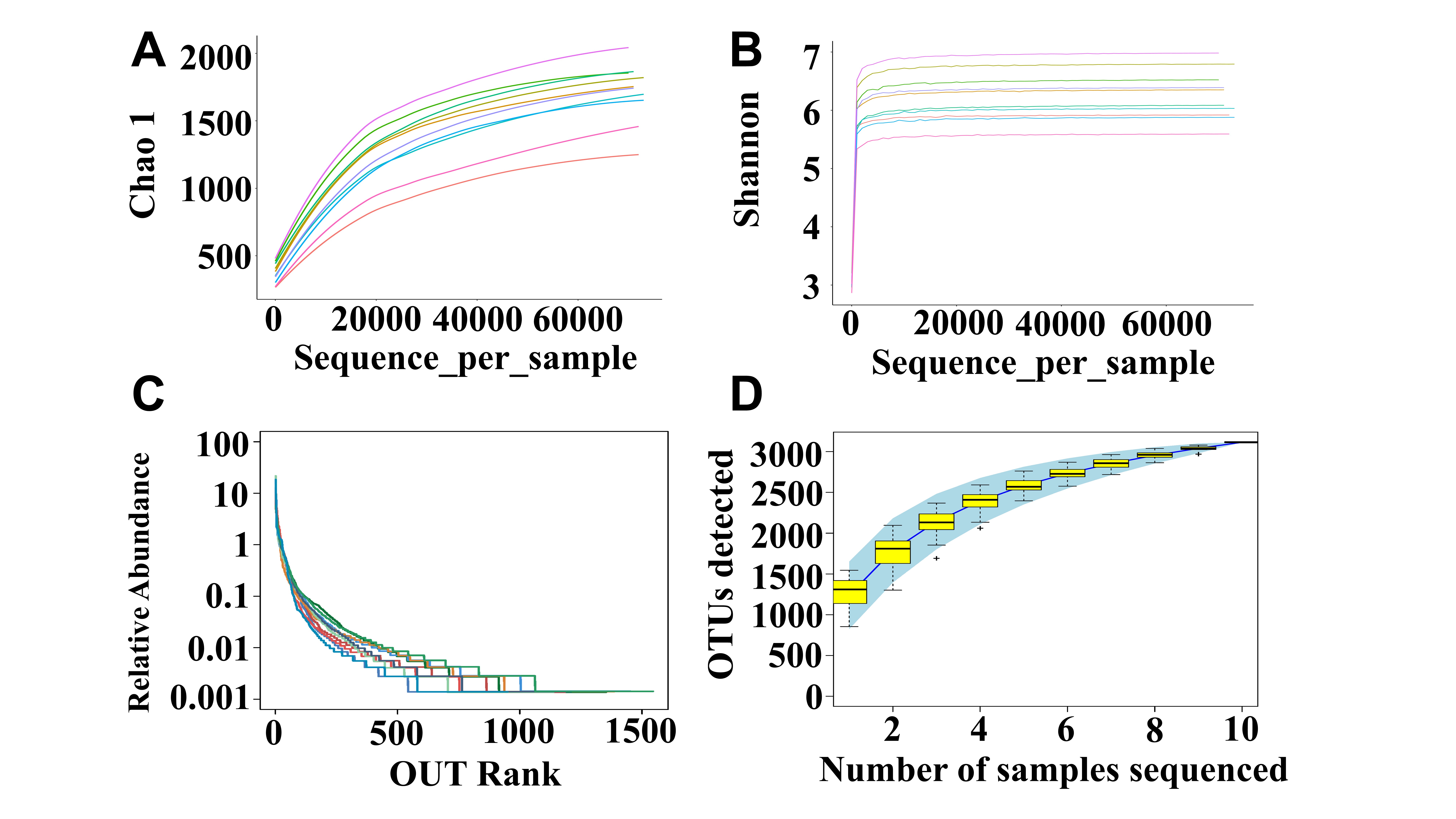
**
